# Computational Cardiac Modeling Reveals Mechanisms of Ventricular Arrhythmogenesis in Long QT Syndrome Type 8: *CACNA1C* R858H Mutation Linked to Ventricular Fibrillation

**DOI:** 10.3389/fphys.2017.00771

**Published:** 2017-10-04

**Authors:** Jieyun Bai, Kuanquan Wang, Yashu Liu, Yacong Li, Cuiping Liang, Gongning Luo, Suyu Dong, Yongfeng Yuan, Henggui Zhang

**Affiliations:** ^1^School of Computer Science and Technology, Harbin Institute of Technology, Harbin, China; ^2^Biological Physics Group, School of Physics and Astronomy, University of Manchester, Manchester, United Kingdom; ^3^Space Institute of Southern China, Shenzhen, China

**Keywords:** *CACNA1C* mutations, L-type calcium channel, Long QT syndrome, Timothy syndrome, ventricular fibrillation, dispersion of repolarization, computational cardiac modeling

## Abstract

Functional analysis of the L-type calcium channel has shown that the *CACNA1C* R858H mutation associated with severe QT interval prolongation may lead to ventricular fibrillation (VF). This study investigated multiple potential mechanisms by which the *CACNA1C* R858H mutation facilitates and perpetuates VF. The Ten Tusscher-Panfilov (TP06) human ventricular cell models incorporating the experimental data on the kinetic properties of L-type calcium channels were integrated into one-dimensional (1D) fiber, 2D sheet, and 3D ventricular models to investigate the pro-arrhythmic effects of *CACNA1C* mutations by quantifying changes in intracellular calcium handling, action potential profiles, action potential duration restitution (APDR) curves, dispersion of repolarization (DOR), QT interval and spiral wave dynamics. R858H “mutant” L-type calcium current (*I*_*CaL*_) augmented sarcoplasmic reticulum calcium content, leading to the development of afterdepolarizations at the single cell level and focal activities at the tissue level. It also produced inhomogeneous APD prolongation, causing QT prolongation and repolarization dispersion amplification, rendering R858H “mutant” tissue more vulnerable to the induction of reentry compared with other conditions. In conclusion, altered *I*_*CaL*_ due to the *CACNA1C* R858H mutation increases arrhythmia risk due to afterdepolarizations and increased tissue vulnerability to unidirectional conduction block. However, the observed reentry is not due to afterdepolarizations (not present in our model), but rather to a novel blocking mechanism.

## Introduction

Congenital long QT syndrome (LQTS) is characterized by an abnormally prolonged QT and high risk of ventricular arrhythmias in susceptible families (Goldenberg et al., 2008). LQTS type 8 (LQT8, Timothy syndrome, TS), a specific subtype of LQTS, is a dysfunction syndrome involving multiple organs, which can manifest as severe QT interval prolongation, T wave alternans, 2:1 atrioventricular block, syndactyly, facial dysmorphism, autistic spectrum disorders, immunodeficiency, severe hypoglycemia, etc. (Splawski et al., 2004, 2005; Etheridge et al., 2011; Gillis et al., 2012). The *CACNA1C* gene encodes Ca_V_1.2 that is a subunit of L-type voltage-dependent calcium channel and gain-of-function mutations in *CACNA1C* have been suggested to be responsible for LQT8 manifestations, but the interplay between *CACNA1C* genotypes and malignant clinical phenotypes is likely complex (Giudicessi and Ackerman, 2013). For instance, the G406R mutation was believed to be the possible cause of TS associated with many extra-cardiac phenotypes, such as syndactyly, cognitive delay, and craniofacial abnormalities (Splawski et al., 2004), while two *de novo* mutations (G406R and G402S) also induced QT prolongation but without syndactyly (Splawski et al., 2005; Frohler et al., 2014; Hiippala et al., 2015). Recently, a handful of other *CACNA1C* mutations were identified in patients exhibiting only modest QT prolongation (Gillis et al., 2012; Boczek et al., 2013, 2015a,b; Fukuyama et al., 2013, 2014; Hennessey et al., 2014; Wemhöner et al., 2015; Landstrom et al., 2016; Sutphin et al., 2016). In particular, Fukuyama et al. identified five novel *CACNA1C* mutations: G1783C, P381S, M456I, A582D, and R858H (Fukuyama et al., 2013, 2014). Patients with the R858H mutation displayed excessive QT prolongation and episodes of ventricular fibrillation (VF). Although functional analysis of R858H mutant channels reveals a significant increase in the L-type calcium current (*I*_*CaL*_), relatively little is known about the pathogenic mechanisms underlying VF in the setting of the “mutant” *I*_*CaL*_.

*I*_*CaL*_ plays a major role in regulating cardiovascular functions because it regulates excitation-contraction coupling (ECC) by triggering the calcium release from the sarcoplasmic reticulum (SR), modulates cellular excitability, and action potential (AP) shape by participating in AP repolarization and is thereby involved in the heart rhythm and contractility (Benitah et al., 2010). Abnormalities in *I*_*CaL*_ due to *CACNA1C* mutations have been suggested as factors contributing to ventricular arrhythmogenesis (Venetucci et al., 2012). In previous simulation studies, it has been shown that changes in *I*_*CaL*_ due to gain-of-function mutations in *CACNA1C* prolongs action potential duration (APD; Faber et al., 2007; Zhu and Clancy, 2007; Yarotskyy et al., 2009; Morotti et al., 2012; Wemhöner et al., 2015; Bai et al., 2016c) linked to early afterdepolarizations (EADs; Sung et al., 2010; Boczek et al., 2015a), and increases SR calcium content which is then responsible for spontaneous calcium release and delayed afterdepolarizations (DADs; Splawski et al., 2005; Thiel et al., 2008; Sung et al., 2010; Yazawa et al., 2011). Although these studies may provide a potential mechanistic link between *CACNA1C* mutations and ventricular arrhythmias, altered AP in single cells cannot be extrapolated directly to reentrant arrhythmias in the human heart, where electrotonic coupling between cardiomyocytes may smooth out electrical heterogeneity between cells. Integrative computational models have been widely used to build a bridge between *CACNA1C* mutations and pro-arrhythmic phenotypes, and these simulated results have shed valuable light on the mechanisms of arrhythmogenesis (Roberts et al., 2012; Bai et al., 2016a). Indeed, our previous models have indicated that changes in *I*_*CaL*_ due to the reduced voltage-dependent inactivation caused by the G1911R mutation in LQT8 extremely prolongs APD and augments dispersion of repolarization (DOR), increasing susceptibility to reentrant arrhythmias (Bai et al., 2016c). By contrast, changes in *I*_*CaL*_ due to the augmented current density caused by the R858H mutation has been suggested to increase the likelihood of VF (Fukuyama et al., 2014), but this link remains to be demonstrated directly. In this study, we focus on the mechanisms by which altered *I*_*CaL*_ caused by a R858H *CACNA1C* mutation promotes and perpetuates ventricular arrhythmia using mathematical modeling.

For this purpose, we modified the Ten Tusscher-Panfilov (TP06) human ventricular cell model (Ten Tusscher et al., 2004, 2006) to incorporate experimental data on the kinetic properties of *I*_*CaL*_ (Fukuyama et al., 2014). We used this model to investigate the electrophysiological consequences of *CACNA1C* mutations in single cells, wave propagation in one-dimensional (1D) ventricular cables, and the onset of spiral waves in two-dimensional (2D) and three-dimensional (3D) models.

In particular, the modified model can reproduce current-voltage (I-V) relationships of *CACNA1C* mutations and prolongation of the QT interval (Fukuyama et al., 2014). We found that *I*_*CaL*_ arising from the *CACNA1C* R858H mutation augments SR calcium content, leading to spontaneous calcium release and afterdepolarizations in single cells, and thereby focal activity in the 1D fiber. The R858H mutation produced electrical heterogeneity within the ventricular wall, amplified the intrinsic spatial DOR, and increased tissue vulnerability to generate unidirectional conduction block facilitating the development of reentry in the transmural ventricular sheet. These simulation data imply that patients with the R858H mutation are at high risk of ventricular arrhythmias.

## Materials and methods

### Model of *I_CaL_*

**Figure 1:**
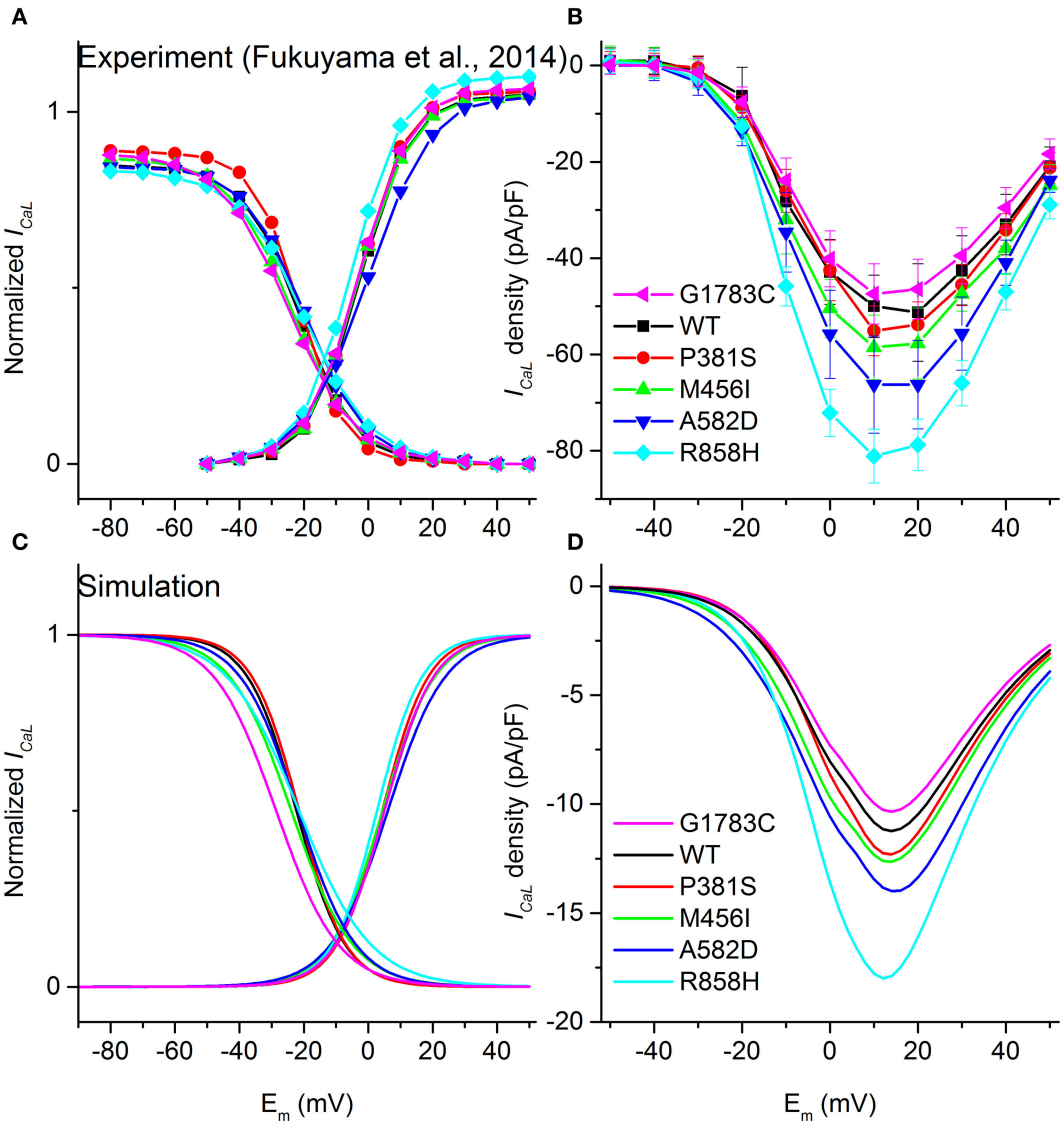
Voltage-dependence of activation and inactivation kinetics for G1783C (Magenta), wild-type (WT, Black), P381S (Red), M456I (Green), A582D (Blue), and R858H (Cyan) conditions. Activation and inactivation curves obtained from experimental data **(A)** as well as simulated results **(C)** are shown. Current-voltage (I–V) relationships obtained from experiments **(B)** and simulations **(D)** are compared.

The equations for *I*_*CaL*_ in the TP06 model (Ten Tusscher and Panfilov, 2006a) were modified to incorporate experimental data on *CACNA1C* mutation-induced changes. First, we determined the modifications to the original *I*_*CaL*_ model to reproduce the behavior of the mutant *I*_*CaL*_ (Figure 1A) during the same voltage-clamp employed in experiments (Fukuyama et al., 2014). Theoretical steady-state activation and inactivation curves which were used to simulate G1783C, wild-type (WT), P381S, M456I, A582D, and R858H *I*_*CaL*_, are shown in Figure 1C. Second, based on experimental I-V relationships (Figure 1B), mathematical models of *I*_*CaL*_ were constructed (formulations are listed in Supplementary Material). This was achieved by simulating the experimental voltage-clamp protocol (Fukuyama et al., 2014) and scaling relative current proportions for G1783C, WT, P381S, M456I, A582D, and R858H conditions. Peak inward G1783C, P381S, M456I, A582D and R858H *I*_*CaL*_ density was, respectively, ~0.86-, ~1.04-, ~1.08-, ~1.19-, ~1.54-fold that for WT *I*_*CaL*_ (Figure 1B). The simulated I-V curves (Figure 1D) matched closely with the experimental observation (Figure 1B).

Upon biophysical analysis of the experimental data on *CACNA1C* mutations (Fukuyama et al., 2014), four major changes to *I*_*CaL*_ were considered. These changes included: *I*_*CaL*_ current densities, steady-state activation curves, voltage-dependent inactivation curves as well as the time constant for the voltage-dependent inactivation. Parameters for *I*_*CaL*_, including the scaling factor of the *I*_*CaL*_ conductance (*CSF*), the midpoint voltage of the voltage-activation curve (*V*_*a, 0.5*_), the slope of the voltage-activation curve (*S*_*a*_), the midpoint voltage of the voltage-inactivation curve (*V*_*ina, 0.5*_), the slope of the voltage-inactivation curve (*S*_*ina*_) and the scaling factor of voltage-inactivation time constant (*TCSF*), were modified to reproduce the experimental I–V relationships (Comparison between simulation and experimental results for *I*_*CaL*_ can be found in Table S1).

### Single cell simulations

The TP06 model, based on human experimental data, was developed to reproduce transmural heterogeneity of electrical properties (Ten Tusscher et al., 2004; Ten Tusscher and Panfilov, 2006a) by changing maximum conductivities of the transient outward potassium channel current (*I*_*to*_) and slow delayed rectifier potassium channel current (*I*_*Ks*_). The *I*_*CaL*_ formulations were the same for endocardial (ENDO), midmyocardial (MCELL) and epicardial (EPI) cell models. The model (Ten Tusscher et al., 2006) was suggested to be suitable for simulating wave dynamics at the tissue and organ levels (Ten Tusscher and Panfilov, 2006b; Ten Tusscher et al., 2009). In 2013, the calcium-induced-calcium release flux (*I*_*rel*_) was modeled as the combination of both SR calcium release and SR calcium leak by Lascano et al. (2013). The same modifications were employed in our previous studies (Bai et al., 2016b, 2017; Liu et al., 2016; formulations are listed in Supplementary Material).

In single cell simulations, APs were elicited by pre-pacing the models for 100 cycles to reach a stable steady state. APD was computed as AP duration at 90% repolarization (APD_90_). Changes in *I*_*CaL*_, sodium-calcium exchanger current (*I*_*NCX*_), *I*_*rel*_, cytoplasmic calcium concentration (*[Ca*^*2*+^*]*_*i*_) and SR calcium concentration (*[Ca*^*2*+^*]*_*SR*_) were used to analyze *CACNA1C* mutations-induced calcium handling. Differences of electrical properties between endocardial- and epicardial- cells (ENDO-EPI), between endocardial- and midmyocardial- cells (ENDO-M) and between epicardial- and midmyocardial- cells (EPI-M) may contribute to transmural electrical heterogeneities in tissues (Zhang et al., 2008). Transmural electrical heterogeneity caused by *CACNA1C* mutations was assessed by quantifying *[Ca*^*2*+^*]*_*i*_ amplitude (*[Ca*^*2*+^*]*_*i*(*m*)_), SR calcium content (*[Ca*^*2*+^*]*_*SR*(*m*)_), *I*_*rel*_ amplitude (*I*_*rel*__(m)_), and APD. The rate dependence of APD was also investigated by using a pacing cycle length (PCL) of 2000, 1000, and 500 ms, respectively.

The maximum slope of the APD restitution (APDR) curve is a main marker for determining whether alternans and spiral breakup will occur (Ten Tusscher and Panfilov, 2006a). We used both the standard S1-S2 and dynamic protocols to determine APDR. For the S1-S2 protocol, 30 S1 stimuli were applied at a PCL of 1,000 ms, and the S2 stimulus was applied at varying diastolic intervals (DI) after the AP evoked by the last S1 stimulus. For the dynamic protocol, a series of 30 stimuli were applied at a PCL of 1,000 ms, after which the PCL was decreased. APDR curves were obtained by plotting APD against DI.

### Multicellular 1D, 2D, and 3D models

**Figure 2:**
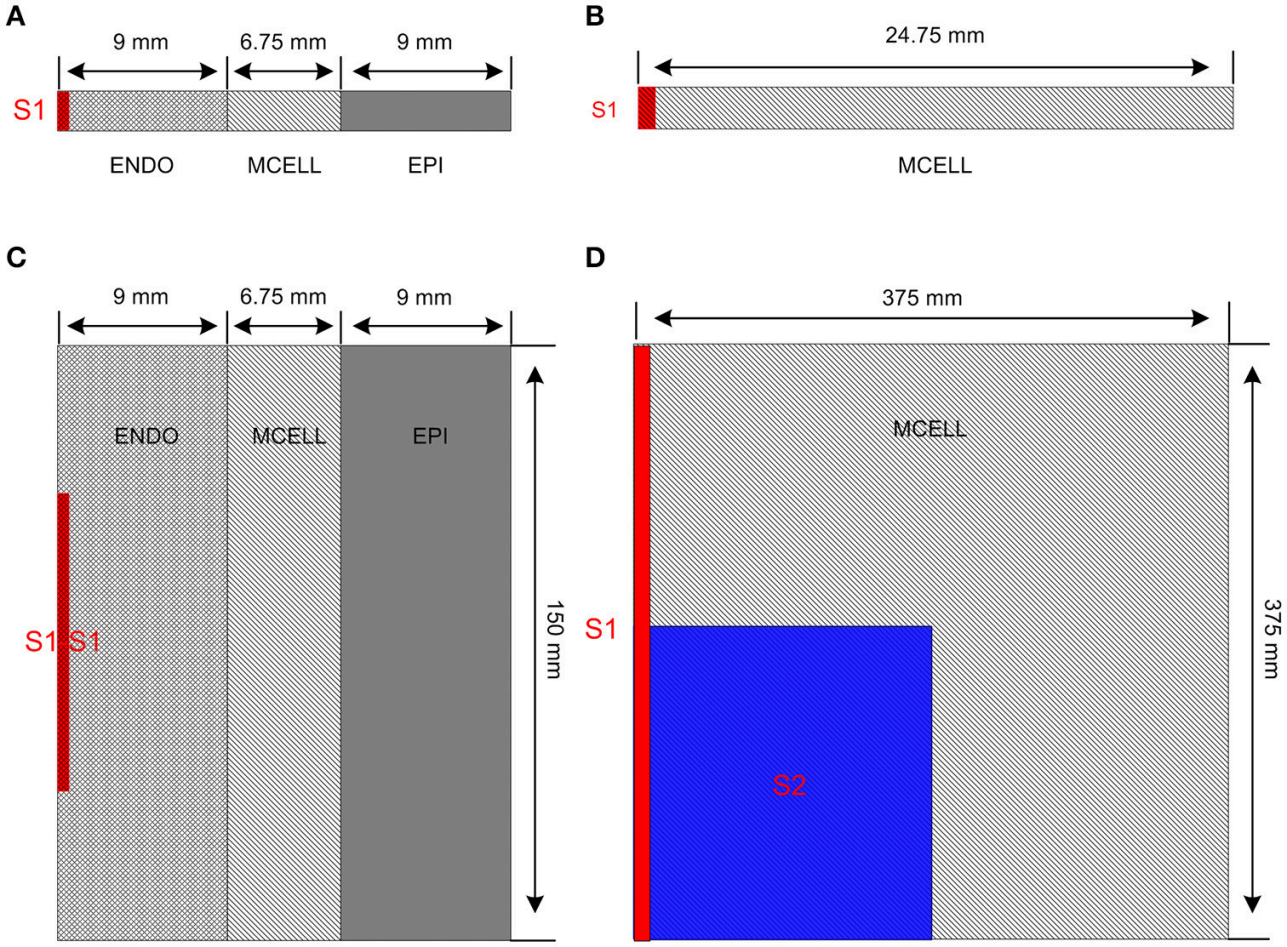
Multicellular one-dimensional (1D) and 2D tissue models. **(A)** A 1D transmural ventricular cable of 24.75 mm which contains 9 mm long endocardial region (ENDO), 6.75 mm long midmyocardial region (MCELL) and 9 mm long epicardial region (EPI). The S1 stimulus is applied to a 0.45 mm ENDO region. **(B)** A 1D homogeneous ventricular cable of 24.75 mm with MCELL cells is constructed and the S1 stimulus is applied to a 0.45 mm region at the end of the cable. **(C)** A 2D transmural ventricular model was constructed by expanding the 1D transmural fiber into a sheet with a length of 150 mm and a width of 24.75 mm. The S1-S1 stimulation is applied to the 0.45 × 75 mm^2^ region at the left side of the ENDO layer. **(D)** A 375 × 375 mm^2^ homogeneous tissue with MCELL cells was developed. Location of S1stimulation (Red) and region of S2 stimulation (Blue) are shown.

Figure 2 shows multicellular tissue models which consist of a 1D transmural ventricular fiber (Figure 2A), a 1D MCELL homogeneous cable (Figure 2B), a 2D transmural ventricular sheet (Figure 2C), and a 2D MCELL tissue (Figure 2D).

The 1D transmural ventricular fiber of length 24.75 mm (Figure 2A), which contained a 9 mm long ENDO region, a 6.75 mm long MCELL region and a 9 mm long EPI region, was constructed to compute a pseudo-ECG and investigate the tissue vulnerability to unidirectional conduction block. The fiber model consisted of 60 ENDO nodes, 45 MCELL nodes and 60 EPI nodes (O'Hara et al., 2011). Following the application of a sequence of 10 conditioning S1 pulses (with amplitude of −40 μA/cm^2^ for 3 ms) applied to a 0.45 mm long ENDO segment at a PCL of 1,000 ms, repolarization characteristics were evaluated by computing repolarization time (RT), DOR, and maximum spatial gradient of APD (MSG). RT was computed as the largest APD in the transmural cable. DOR was measured as the difference between the largest and smallest APD of cells in the fiber. Spatial gradient of APD (SG) along the transmural cable was calculated as changes of APD per millimeter. In addition, the pseudo-ECG was obtained by using the method of Gima and Rudy (Gima and Rudy, 2002; formulations are listed in Supplementary Material). QT interval, T-wave width and T-wave amplitude were quantified to examine the changes in the ECG due to *CACNA1C* mutations. QT interval was estimated as the time interval between the Q-wave onset and the T-wave end, T-wave width was computed as the peak and the end of the T-wave, and T-wave amplitude was defined as the peak voltage of the T-wave. The T-wave end was determined by the intersection point of the baseline (*y* = 0 mV) and the T wave. The inducibility of unidirectional conduction block for each mutant *CACNA1C* was also quantified by computing the maximum PCL (MPCL) that produced 2:1 block.

A 1D MCELL cable of length 24.75 mm (Figure 2B) was used to measure conduction velocity (CV) by calculating the time Δ*t* for the wavefront to propagate from *x* − Δ*x* to *x* + Δ*x*, defining CV = 2 Δ*x/*Δ*t*. To examine whether focal activity in the 1D cable was induced at a PCL of 500 ms, solitary planar waves were initiated by applying S1 pulses (with the same size, strength and duration as the one used for the 1D transmural simulation).

A transmural ventricular sheet (Figure 2C), which was constructed by expanding the 1D transmural fiber into a sheet with a length of 24.75 mm and a width of 150 mm, was developed to examine tissue vulnerability to the initiation of reentrant waves. A S1-S1 stimulation (with the same strength and duration as the one used for the 1D simulations) was applied to a region of 0.45 × 75 mm^2^, located at the center of the left ENDO boundary.

A 2D MCELL tissue model of 375 × 375 mm^2^ (Figure 2D) was developed to investigate effects of *CACNA1C* mutations on the spatiotemporal behavior of spiral waves. Spiral waves were induced by a standard S1–S2 stimulation. A plane wave was initiated by applying the S1 stimulus to the left side of the domain (0.75 × 375 mm^2^). Once the plane wave had passed over the first half of the domain, the S2 stimulus was applied to the first quarter of the domain so that a spiral wave was produced. AP was recorded from the representative point (*x* = 187.5 mm, *y* = 187.5 mm) and the fundamental frequency was obtained from the power spectra of the AP.

For the 3D model, simulations were performed using an anatomical human ventricular geometry developed in our previous studies (Bai et al., 2015, 2016c). It has a spatial resolution of 0.5 mm with ~6.38 million cells in total. For both left and right ventricles, the tissue was segmented into distinctive ENDO, MCELL, and EPI layers with similar contiguous figurations in the transmural wall as in the 1D transmural ventricular fiber model. The S1-S1 stimulation was applied to a 2.5 mm wide region of the ENDO layer. For the purposes of the present study, the 3D anatomical model was assumed to be electrically homogeneous.

### Numerical methods

The cell models were incorporated into a parabolic partial differential equation (PDE) to construct mono-domain models of cardiac electrophysiology to describe the reaction-diffusion system in simulating cardiac dynamics (Clayton and Panfilov, 2008). The governing equation is

(1) $$\begin{array}{l} {\text{C}}_{\text{m}}\frac{\partial {\text{V}}_{\text{m}}}{\partial \text{t}}=\text{D}\nabla^{2}{\text{V}}_{\text{m}}-{\text{I}}_{\text{i}\text{o}\text{n}} \end{array}$$

where ***C***_*m*_ = 1 μF*/*cm^2^ is the capacitance, ***D*** is the effective diffusion constants, and ***I***_*ion*_ is the total transmembrane current.

We used a forward-Euler method for marching, with a time step (Δ*t*) of 0.02 ms and a space step (Δ*x* = Δ*y* = Δ*z*), to solve the PDEs (Equation 1). The value of *D* is set to be 0.0385 mm^2^/ms for simulating excitation waves in the 1D transmural ventricular fiber, the 1D homogeneous cable, the 2D transmural ventricular sheet and the 3D ventricular model. The spatial resolution in 1D, 2D and 3D models are as follows: 1D ventricular model: Δ*x* = 0.15 mm; 2D transmural ventricular model: Δ*x* = Δ*y* = 0.15 mm; 3D ventricular model: Δ*x* = Δ*y* = Δ*z* = 0.5 mm. For the 2D MCELL ventricular model, *D* is set to be 0.154 mm^2^/ms and the spatial resolution is chosen to be 0.25 mm to investigate the role of APDR in the occurrence of electrical instability (Ten Tusscher et al., 2004; Vandersickel et al., 2014; Nayak and Pandit, 2015; Zimik et al., 2015; Nayak et al., 2017). We also used Neumann (i.e., no-flux) boundary conditions (Clayton and Panfilov, 2008). Simulations were carried out on a 64-bit Intel core i7-3930K CPU system with 64 GB memory. Efficient parallelization was implemented using GPU acceleration (Bai et al., 2015). Although different time, space and diffusion coefficient were used, solutions to the mono-domain model with isotropic diffusion fulfilled the stability criterion: (i.e., *D*Δ*t/* Δ*x*^2^ < 1/2; Clayton and Panfilov, 2008).

## Results

### Effects of *CACNA1C* mutations on intracellular calcium handling and action potential

**Figure 3:**
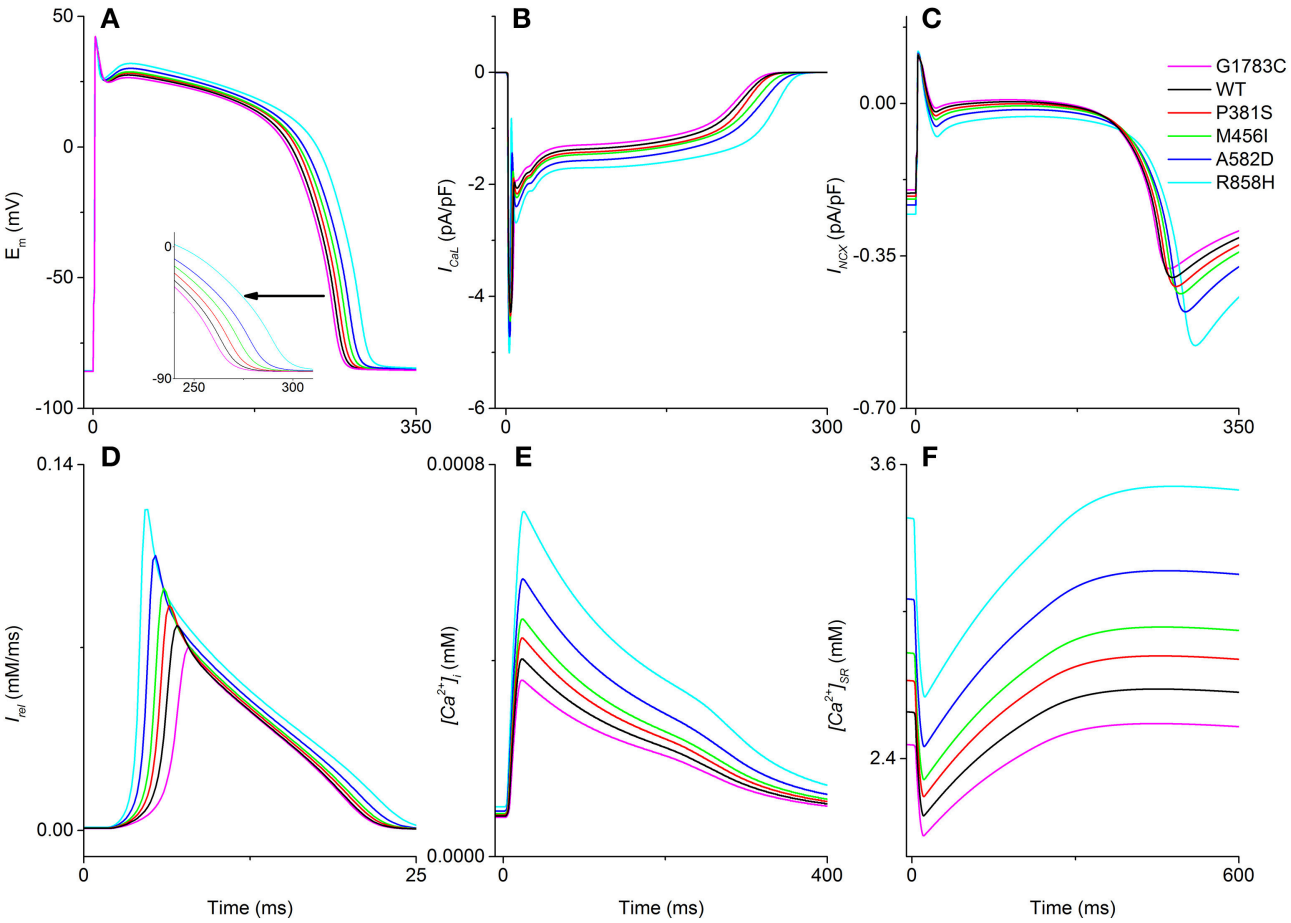
Intracellular calcium handling and action potential. For endocardial cells, time courses of the membrane potential (E_m_, **A**), the L-type calcium current (*I*_*CaL*_, **B**), the sodium-calcium exchanger current (*I*_*NCX*_, **C**), calcium induced calcium release flux (*I*_*rel*_, **D**), cytoplasmic calcium concentration (*[Ca*^*2*+^*]*_*i*_, **E**) and sarcoplasmic reticulum (SR) calcium concentration (*[Ca*^*2*+^*]*_*SR*_, **F**) in the G1783C (Magenta), wild-type (WT, Black), P381S (Red), M456I (Green), A582D (Blue), and R858H (Cyan) conditions are shown. The pacing cycle length (PCL) used in these simulations is 1,000 ms. Action potential duration, *I*_*NCX*_, *I*_*rel*_, *[Ca*^*2*+^*]*_*i*_, and *[Ca*^*2*+^*]*_*SR*_ augment with an increase in *I*_*CaL*_. Changes in action potential duration are indicated by an enlargement of the **(A)**.

*I*_*CaL*_ arising from the Ca_V_1.2 mutations altered intracellular calcium handling and prolonged AP as shown in Figure 3A. For ENDO cells, the *I*_*CaL*_ amplitude was increased progressively from 4.12 pA/pF in the G1783C condition (Magenta) to 4.28 pA/pF (WT, Black), 4.35 pA/pF (P381S, Red), 4.44 pA/pF (M456I, Green), 4.71 pA/pF (A582D, Blue), and 5.0 pA/pF (R858H, Cyan), respectively (Figure 3B). Altered intracellular calcium handling resulted from increased *I*_*CaL*_ was shown by the time courses of *I*_*NCX*_(Figure 3C), *I*_*rel*_ (Figure 3D), *[Ca*^*2*+^*]*_*i*_ (Figure 3E) and *[Ca*^*2*+^*]*_*SR*_(Figure 3F). Changes in *[Ca*^*2*+^*]*_*i*(*m*)_, *[Ca*^*2*+^*]*_*SR*(*m*)_, and *I*_*rel*__(m)_ were related to an increase in *I*_*CaL*_ (changes are summarized in Table S2). Increased *I*_*CaL*_ during the AP plateau triggered a large *I*_*rel*_, leading to cytoplasmic calcium overload and APD prolongation. The measured APD was 264.4 ms (G1783C), 267.8 ms (WT), 271.8 ms (P381S), 277 ms (M456I), 282.6 ms (A582D), and 294 ms (R858H), respectively.

Simulations of transmural electrical heterogeneity were performed for each mutation, producing APs of ENDO, MCELL, and EPI cells. *[Ca*^*2*+^*]*_*i*(*m*)_, *[Ca*^*2*+^*]*_*SR*(*m*)_, *I*_*rel*(*m*)_, and APD were used to quantify electrical properties of the different cell types (listed in Table S2). The electrical differences associated with each mutant *CACNA1C* for ENDO-EPI, ENDO-M as well as EPI-M are summarized in Table S3. There were small electrical differences for ENDO-EPI, whilst large electrical heterogeneities were found for EPI-M and ENDO-M. For one “mutant” *I*_*CaL*_ tested (R858H), *[Ca*^*2*+^*]*_*SR*(*m*)_ of ENDO-EPI (~0.1 mM) was smaller than that of EPI-M (~0.64 mM) as well as ENDO-M (~0.74 mM). Among in these mutations, the Ca_V_1.2 R858H mutation with the largest effect on electrophysiological heterogeneity was present. For the *[Ca*^*2*+^*]*_*SR*(*m*)_, the ENDO-M difference for the R858H condition was larger (~0.74 mM) than the one (~0.64 mM) produced by the presence of the WT *I*_*CaL*_, while other mutations caused changes ranging from ~0.59 mM to ~0.69 mM. Other characteristics of transmural heterogeneity are listed in Table S3, which demonstrate that the largest electrophysiological heterogeneity is with the R858H condition.

### Effects of *CACNA1C* mutations on induction of afterdepolarizations

**Figure 4:**
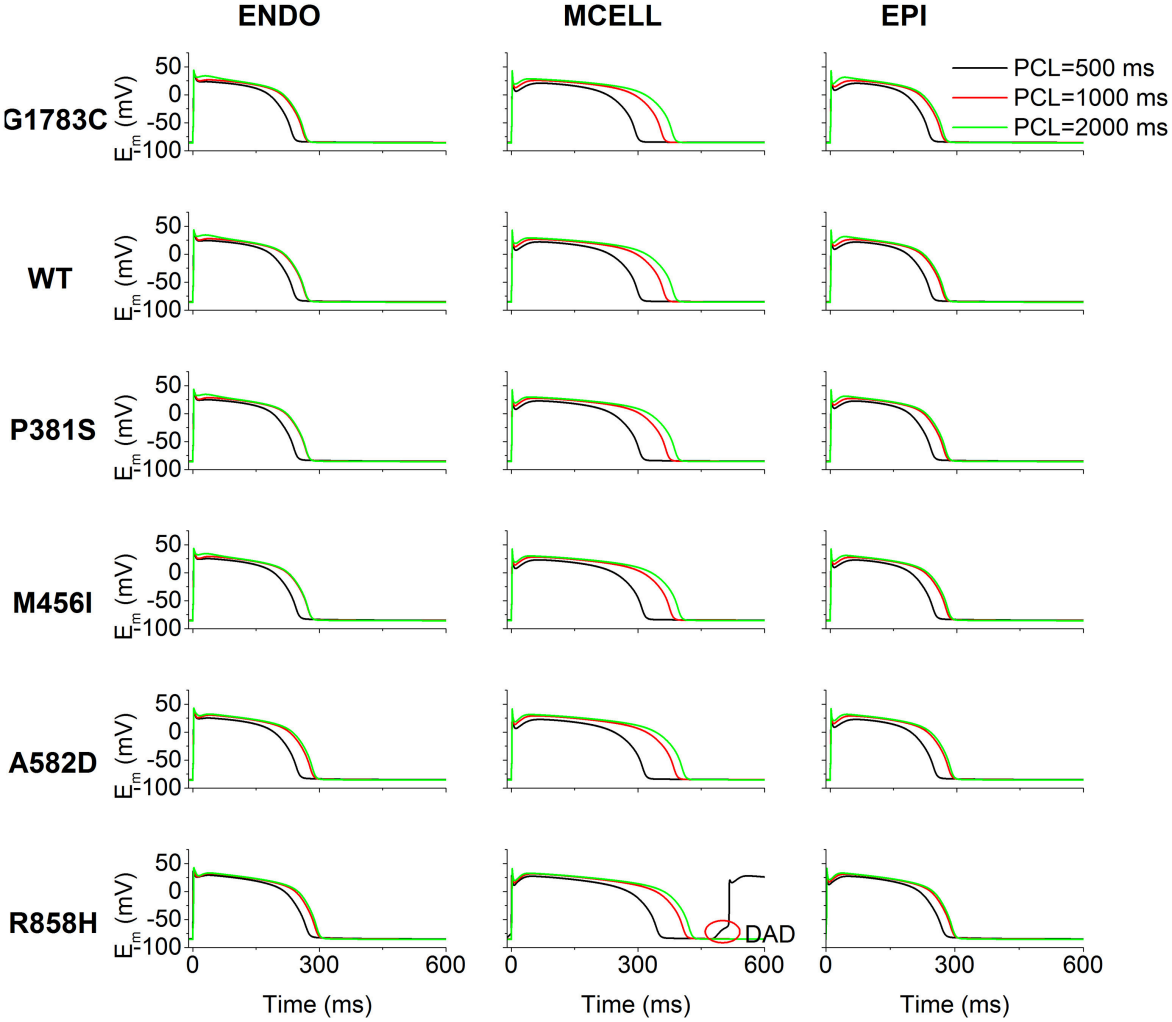
Simulated action potentials (E_m_) for the Ten Tusscher-Panfilov (TP06) human ventricular cell model at different pacing cycle lengths (PCL) under G1783C, wild-type (WT), P381S, M456I, A582D, and R858H conditions. Endocardial (ENDO, **left column**), midmyocardial (MCELL, **middle column**) and epicardial (EPI, **right column**) action potentials at the PCL of 500 (Black), 1,000 (Red), and 2,000 ms (Green), respectively. Action potential duration abbreviates with a decrease in PCL. For each mutation, action potential duration of MCELL cells is longer than that of other cells. Among in these mutations, when the PCL is 500 ms for R858H, delayed afterdepolarizations (DAD, marked with a red circle) were triggered in MCELL cells.

The effects of *I*_*CaL*_ associated with *CACNA1C* mutations on AP shape are shown in Figure 4. Cell simulations were conducted by increasing the pacing frequency from 0.5 to 2 Hz [corresponding to PCL of 2,000 ms (Green), 1,000 ms (Red) as well as 500 ms (Black)] and the measured APDs for G1783C, WT, P381S, M456I, A582D, and R858H cells are listed in Table S4. For the ENDO (Figure 4, left column), MCELL (Figure 4, middle column) and EPI (Figure 4, right column) cells, APD_90_ was abbreviated with PCL and no afterdepolarizations were triggered under the G1783C, WT, P381S, M456I, and A582D conditions. However, under the R858H condition, a DAD in the MCELL cells was induced, but no ectopic beats in the EPI and ENDO cells were observed, when the PCL was 500 ms.

**Figure 5:**
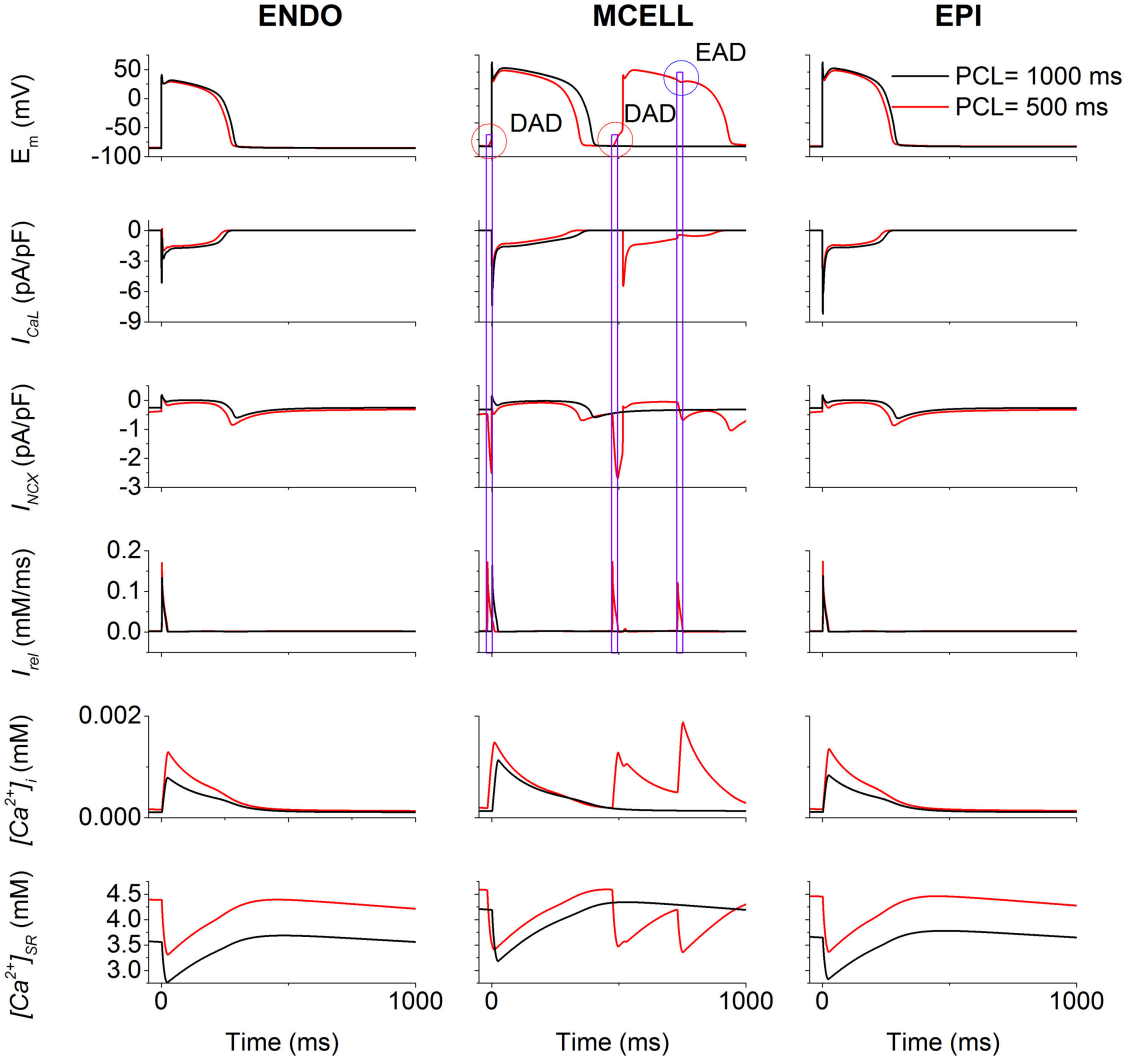
Effects of the R858H L-type calcium current (*I*_*CaL*_) on the induction of afterdepolarizations. For endocardial (ENDO, **left column**), midmyocardial (MCELL, **middle column**) and epicardial (EPI, **right column**) cells, time courses of membrane potential (E_m_), underlying L-type calcium current (*I*_*CaL*_), the sodium-calcium exchanger current (*I*_*NCX*_), calcium induced calcium release flux (*I*_*rel*_), cytoplasmic calcium concentration (*[Ca*^*2*+^*]*_*i*_) as well as sarcoplasmic reticulum (SR) calcium concentration (*[Ca*^*2*+^*]*_*SR*_) at the pacing cycle length (PCL) of 500 (Red) and 1,000 ms (Black), respectively. Among different cell types, in MCELL cells early afterdepolarizations (EAD, marked with a blue circle) and delayed afterdepolarizations (DAD, marked with a red circle) occurred at the PCL of 500 ms. These afterdepolarizations were triggered by *I*_*rel*_ via *I*_*NCX*_.

To illustrate afterdepolarizations generating events, the time courses of AP, *I*_*CaL*_, *I*_*NCX*_, *I*_*rel*_, *[Ca*^*2*+^*]*_*i*_, and *[Ca*^*2*+^*]*_*SR*_ for the ENDO (Figure 5, left column), MCELL (Figure 5, middle column) and EPI (Figure 5, right column) cells at PCL of 500 ms (Red) and 1,000 ms (Black) are shown. As can be seen, *I*_*CaL*_ arising from the R858H mutation contributed to an increase in *[Ca*^*2*+^*]*_*SR*_, which, consequently, enhanced spontaneous *I*_*rel*_, accompanied by an inward *I*_*NCX*_ that depolarized the cell. Subsequently, EADs (marked with a blue circle) and DADs (marked with red circles) were triggered in R858H-MCELL cells. Compared with R858H-ENDO and R858H-EPI cells, spontaneous *I*_*rel*_ frequency was higher in R858H-MCELL cells. Additionally, spontaneous *I*_*rel*_ occurred during repolarization and therefore led to the EADs, however, DADs were induced by spontaneous *I*_*rel*_ after repolarization. In addition, the APD (≈421.2 ms) of the DAD-induced AP was larger than that (348.2 ms) of the R858H-MCELL cells at a PCL of 500 ms, indicating QT interval prolongation at rapid heart rates.

### Effects of *CACNA1C* mutations on APD restitution

**Figure 6:**
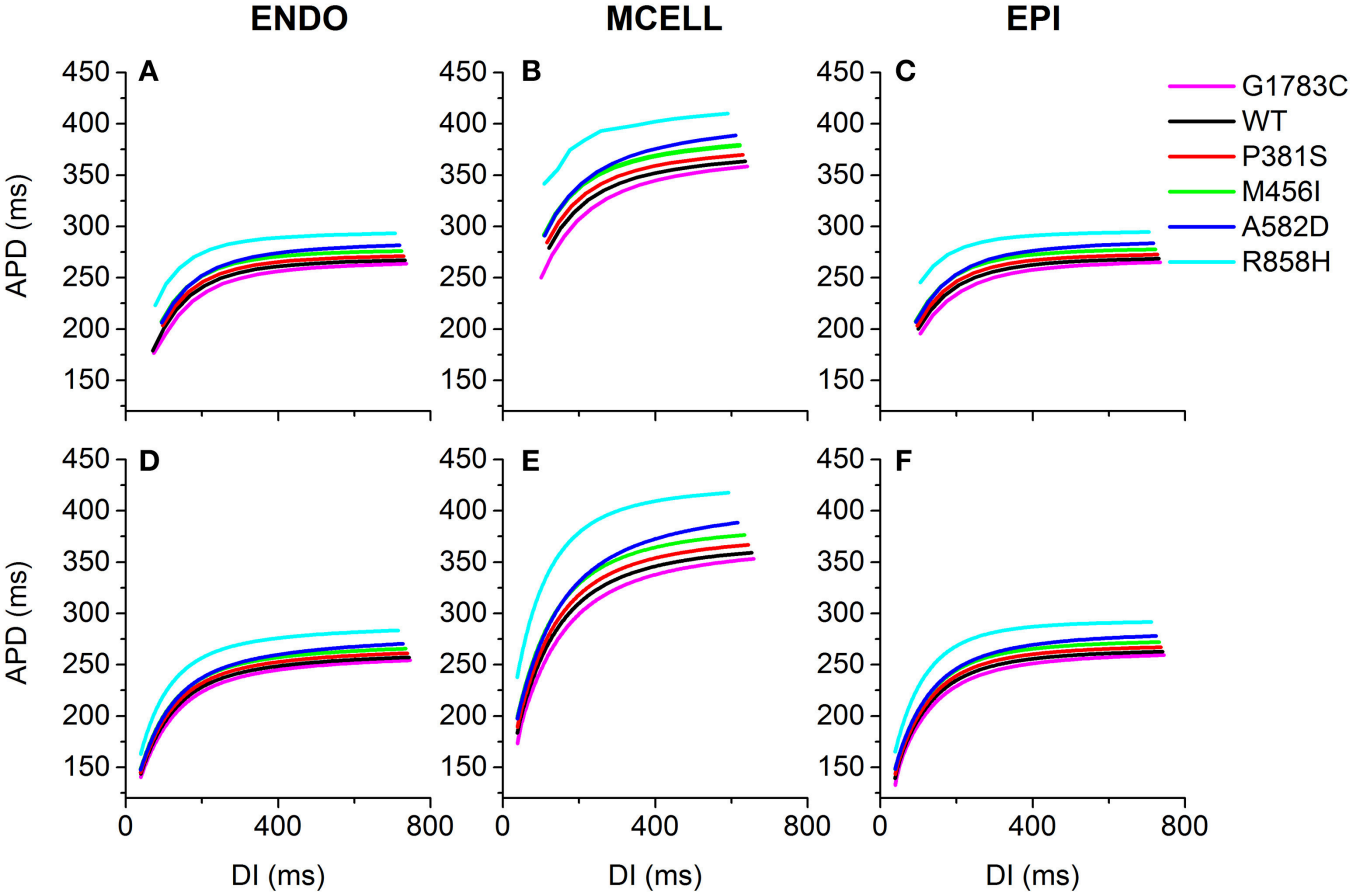
Single-cell action potential duration (APD) restitution (APDR) curves for the Ten Tusscher-Panfilov (TP06) human ventricular cell model. **(A–C)** APDR curves obtained using a dynamic restitution protocol for the endocardial (ENDO), epicardial (EPI) and midmyocardial (MCELL) cells in the G1783C (Magenta), wild-type (WT, Black), P381S (Red), M456I (Green), A582D (Blue), and R858H (Cyan) conditions. **(D–F)** Similar dynamic restitution curves as in **(A–C)**, but these APDR curves obtained using a S1-S2 restitution protocol for a pacing cycle length (PCL) of 1,000 ms for the six different settings. APD is plotted against diastolic interval (DI). For each cell type, curves from bottom to top are for the G1783C mutation, for the WT condition, for the P381S mutation, for the M456I mutation, for the A582D mutation and for the R858H mutation, respectively. Among in these mutations, the R858H mutation obviously shifts APDR curves upwards.

The effects of the mutant *I*_*CaL*_ on ventricular APDR are shown in Figure 6. The APD reduction was rate-dependent for ENDO (Figures 6A,D), EPI (Figures 6B,E) and MCELL (Figures 6C,F) cells. Across the range of DIs tested, the measured APD was larger for the R858H (Cyan) condition than for the G1783C (Magenta), WT (Black), P381S (Red), M456I (Green), and A582D (Blue) conditions. For the R858H settings, the maximum restitution slope of 2 for MCELL cells (Figure 6E) was larger than those for ENDO (Figure 6D, 1.35) as well as EPI cells (Figure 6F, 1.5). For MCELL cells, the computed APDR slopes of MCELL cells (Figure 6E) were 2 (G1783C), 1.8 (WT), 1.7 (P381S), 1.7 (M456I), 1.7 (A582D), and 2 (R858H), respectively. Details of maximum APDR slopes for three cell types can be found in Table S5.

### Effects of *CACNA1C* mutations on ECG

**Figure 7:**
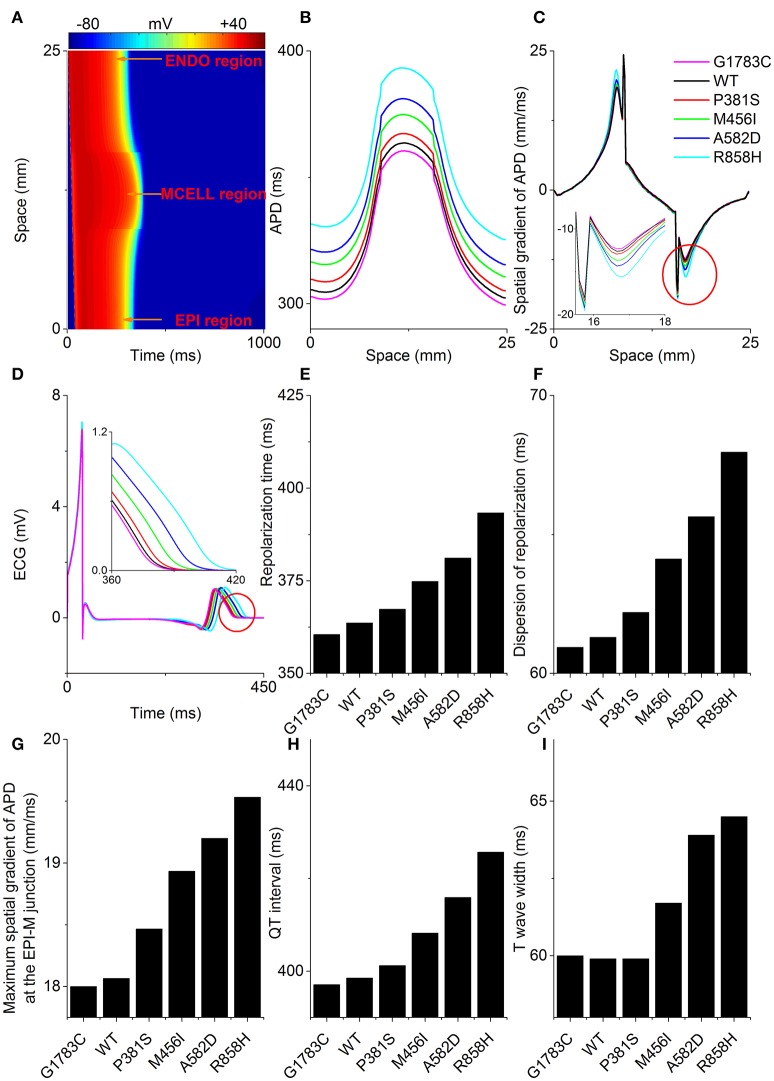
Space-time plot of action potential propagation and the computed pseudo-ECG. **(A)** Color mapping of membrane potential of cells along the one-dimensional (1D) strand from blue (−86 mV) to red (+42 mV). Space runs from the endocardial (ENDO) region to the midmyocardial (MCELL) and epicardial (EPI) regions. Spatial distribution of action potential duration (APD, **B**), spatial gradient of APD **(C)**, and pseudo-ECGs **(D)** corresponding to the G1783C (Magenta), wild-type (WT, Black), P381S (Red), M456I (Green), A582D (Blue), and R858H (Cyan) conditions, respectively. Computed repolarization time (RT, **E**), dispersion of repolarization (DOR, **F**), maximum spatial gradient of APD (MSGA) at the epicardial- and midmyocardial- (EPI-M) junction **(G)**, QT interval **(H)** and T wave width **(I)** are shown. From bottom to top, curves for the spatial distribution of APD are for the G1783C mutation, for the WT condition, for the P381S mutation, for the M456I mutation, for the A582D mutation and for the R858H mutation, respectively. Changes in the spatial gradient of APD at the EPI-M junction (marked with a red circle) are indicated by an enlargement of the **(C)**. Changes in the T wave (marked with a red circle) are indicated by an enlargement of the **(D)**. RT, DOR, MSGA at the EPI-M junction and QT interval augment with an increase in APD. T wave widths show no apparent differences between G1783C, WT and P381S.

To examine the manifestation of the temporal and spatial dispersion of AP in the pseudo-ECG, AP propagation (Figure 7A), spatial distribution of APD (Figure 7B), spatial gradient of APD (Figure 7C) and the computed pseudo-ECG (Figure 7D) of 1D transmural strand at a PCL of 1,000 ms for each “mutant” *I*_*CaL*_ are shown. Repolarization time (Figure 7E), DOR (Figure 7F), the maximum spatial gradient of APD at the EPI-M junction (Figure 7G), QT interval (Figure 7H), and T wave width (Figure 7I) were computed for the G1783C, WT, P381S, M456I, A582D, and R858H conditions. As can be seen in Figure 7D, the QT interval was prolonged from 397.1 ms (G1783C) to 398.5 ms (WT), 401.2 ms (P381S), 408.2 ms (M456I), 415.9 ms (A582D), and 425.7 ms (R858H), respectively (Figure 7H). The QT interval prolongation underlying increased APD in single cells has been linked to repolarization time (RT; Gima and Rudy, 2002). Therefore, the effects of the mutant *I*_*CaL*_ on RT were examined. The computed RT was 360.48 ms (G1783C), 363.6 ms (WT), 367.34 ms (P381S), 374.84 ms (M456I), 381.14 ms (A582D), and 393.32 ms (R858H), respectively (Figure 7E). The QT prolongation was consistent with the prediction of RT. Moreover, the APD of MCELL cells with the longest repolarization time was increased from 359 ms (G1783C) to 364.2 ms (WT), 370.4 ms (P381S), 379.6 ms (M456I), 389.4 ms (A582D), and 410.8 ms (R858H), respectively (listed in Table S6). The QT interval prolongation can be attributed to *I*_*CaL*_ due to *CACNA1C* mutations that influence APD in single cells and ventricular RT in ventricular tissues.

In addition, changes in the T wave were examined. As is shown in Figure 7H, the T-wave width was 60 ms (G1783C), 59.9 ms (WT), 59.9 ms (P381S), 61.7 ms (M456I), 63.9 ms (A582D), and 64.5 ms (R858H), respectively (Figure 7I). The augmented T-wave width has been attributed to an increase in DOR (Antzelevitch et al., 1998), therefore the effects of the mutant *I*_*CaL*_ on APD dispersion in the transmural strand were also investigated. The computed DOR was enhanced from 60.94 ms (G1783C) to 61.3 ms (WT), 62.2 ms (P381S), 64.12 ms (M456I), 65.64 ms (A582D), and 67.96 ms (R858H; Figure 7F). In addition, it has been suggested that DOR is a marker of electrical heterogeneity in APD (Antzelevitch, 2001). Comparatively, the maximal APD difference for EPI-M was 93 ms (G1783C), 94.8 ms (WT), 97 ms (P381S), 101 ms (M456I), 104.8 ms (A582D), and 115.2 ms (R858H), respectively (summarized in Table S3). The APD heterogeneity induced by *CACNA1C* mutations may contribute to DOR and thereby to T-wave width.

**Figure 8:**
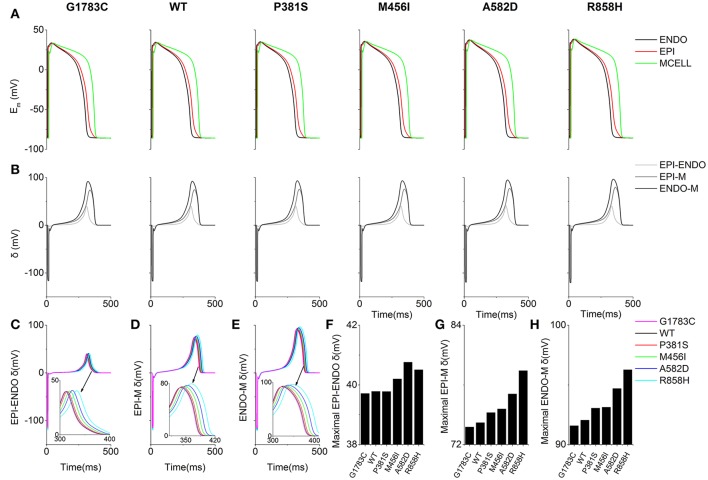
Membrane potential heterogeneity (δ) for EPI-ENDO (between epicaridal and endocardial cells), EPI-M (between epicaridal and midmyocardial cells) and ENDO-M (between endocaridal and midmyocardial cells) in a one-dimensional (1D) transmural ventricular cable. **(A)** Endocardial (ENDO, Black), epicardial (EPI, Red), and midmyocardial (MCELL, Green) membrane potentials (E_m_) for the G1783C, wild-type (WT), P381S, M456I, A582D and R858H conditions. **(B)** EPI-ENDO (Light Gray), EPI-M (Gray) and ENDO-M δ (Dark Gray). **(C–E)** Superimposed EPI-ENDO, EPI-M and ENDO-M δ. **(F–H)** Absolute maximum EPI-ENDO, EPI-M, and ENDO-M δ.

Similarly, T-wave amplitudes were calculated. The T-wave amplitude was 1.051 mV (G1783C), 1.053 mV (WT), 1.056 mV (P381S), 1.075mV (M456I), 1.087 mV (A582D), and 1.104 mV (R858H), respectively (listed in Table S6). Compared with the WT condition, there was no evident difference in T-wave amplitude. Changes of T-wave amplitude can be attributed to altered temporal and spatial gradients in membrane potential (Gima and Rudy, 2002; Zhang et al., 2008). Therefore, the APD gradient (Figure 7C) and the membrane potential gradient (δ, Figure 8) were computed. Figure 8A shows simulated ENDO (Black), EPI (Red), MCELL (Green) APs for each mutant condition whilst Figure 8B shows corresponding time-course plots of δ for EPI-ENDO (Light Gray), EPI-M (Gray), and ENDO-M (Dark Gray). The maximal δs for EPI-M (Figure 8D) and ENDO-M (Figure 8E) were greater than that for EPI-ENDO (Figure 8C). This was consistent with the prediction of electrical heterogeneities of AP in single cells (summarized in Table S3). There was no significant change in the maximum EPI-ENDO δ between these mutations (Figure 8F). However, the maximum EPI-M δ was 73.8 mV (G1783C), 74.2 mV (WT), 75.2 mV (P381S), 75.6 mV (M456I), 77.1 mV (A582D), and 79.4 mV (R858H), respectively (Figure 8G), and the maximum ENDO-M δ was 91.59 mV (G1783C), 92.06 mV (WT), 93.06 mV (P381S), 93.14 mV (M456I), 94.69 mV (A582D), and 96.27 mV (R858H), respectively (Figure 8H). Also, the maximum spatial gradient (MSG) of APD at the EPI-M junction was 18 ms/mm (G1783C), 18.1 ms/mm (WT), 18.5 ms/mm (P381S), 18.9 ms/mm (M456I), 19.2 ms/mm (A582D), and 19.5 ms/mm (R858H), respectively (Figure 7G). Changes in MSG at the EPI-M junction and maximal EPI-M δ were consistent with the altered T-wave amplitude.

Taken together, the ECG phenotypes were dependent on changes of AP caused by altered *I*_*CaL*_ due to *CACNA1C* mutations in single cells and the longest QT interval was obtained under the R858H condition (listed in Table S6).

### Effects of *CACNA1C* mutations on action potential propagation

**Figure 9:**
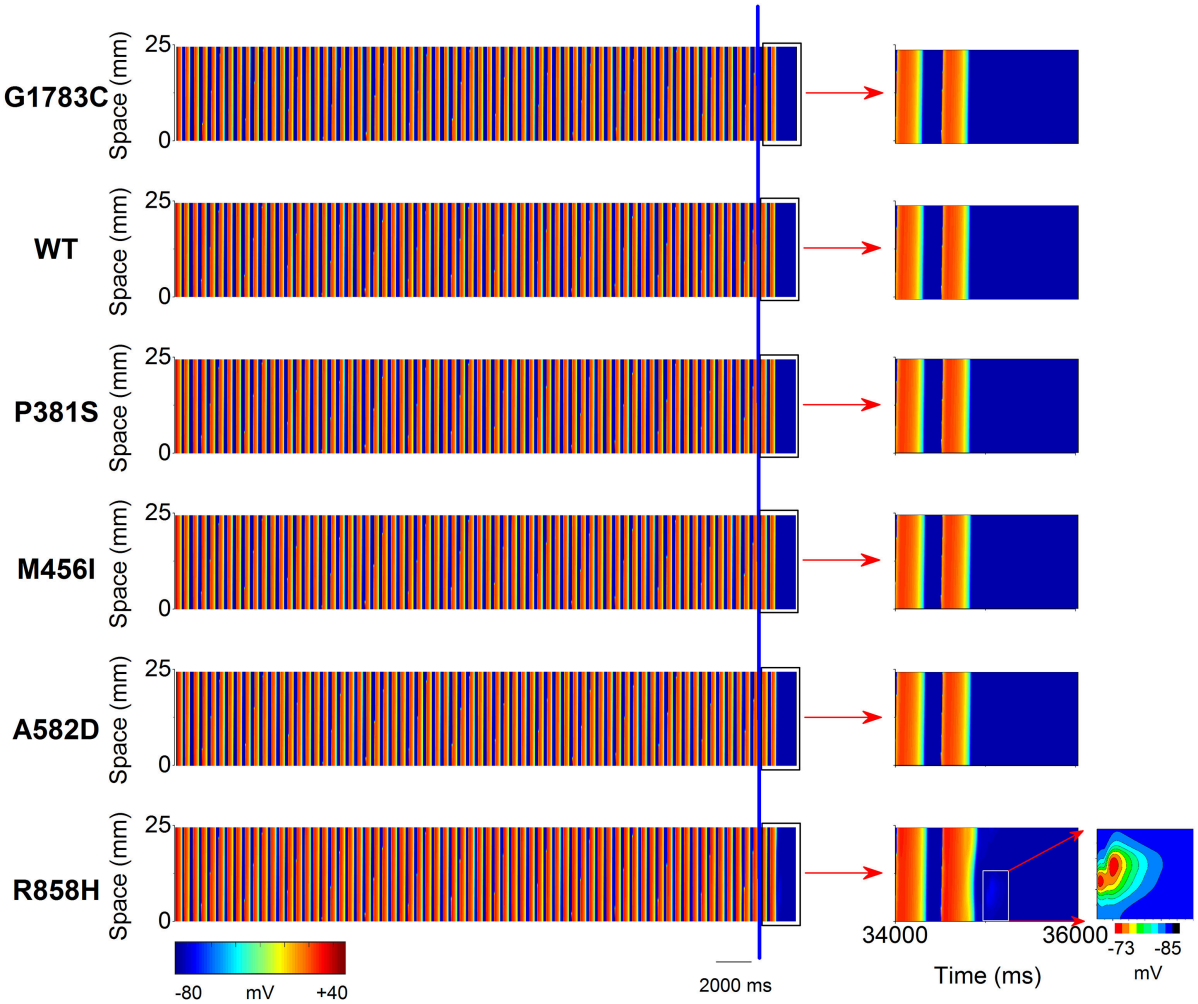
Space-time plots of midmyocardial action potential propagation in a one-dimensional (1D) homogeneous ventricular cable at the pacing cycle length (PCL) of 500 ms. Color mappings of membrane potential (**left column**) are shown under G1783C, wild-type (WT), P381S, M456I, A582D, and H858R conditions, respectively. The last two beats (the part on the right of the blue line) obtained from 34,000 to 36,000 ms (**right column**, marked with a black rectangle). A local region (marked with a white rectangle) close to the pacing site exhibiting a subthreshold delayed afterdepolarization (DAD) with no premature ventricular complex (PVC) under the R858H condition. Changes in membrane potential of the local region are indicated by an enlargement of the marked zone. Color mapping of membrane potential from blue (−85 mV) to red (−73 mV).

To investigate the cellular level conditions required for afterdepolarizations to trigger a premature ventricular complex (PVC) at the multicellular tissue level, a 24.75-mm long MCELL strand model was constructed and space-time plots of AP propagation in the 1D homogeneous cable at the PCL of 500 ms are shown in Figure 9. For the G1783C, WT, P381S, M456I and A582D conditions, no focal activity was observed whilst a R858H-mediated focal activity (marked with a white rectangle) was triggered at Time = 35,000 ms. Although a DAD mediated-AP was induced in MCELL cells (Figure 5, middle column), such simulations may not necessarily reflect the situation for intact tissue, in which electrical coupling occurs between cells and may smooth out these electrical differences. Thus, the propagation of the DAD mediated-AP was not observed (Figure S1) and thereby no PVC occurred under the R858H condition.

**Figure 10:**
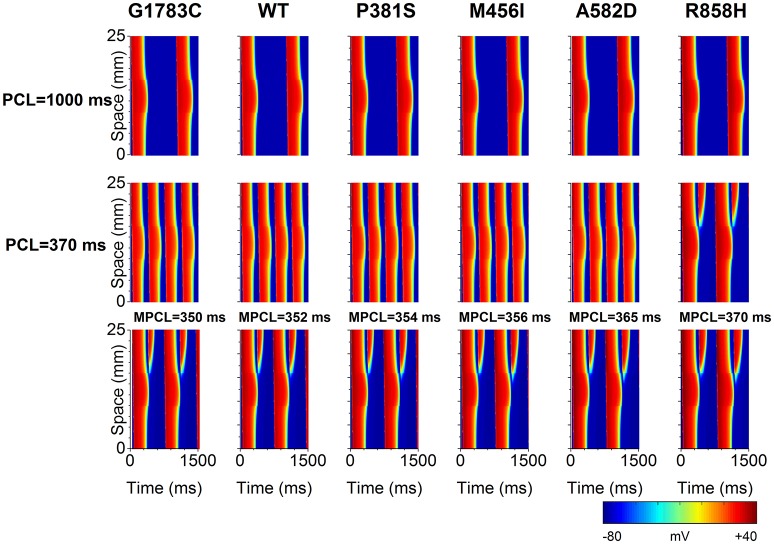
Space-time plots of action potential propagation in a one-dimensional (1D) transmural ventricular cable at different pacing cycle lengths (PCL). At PCL of 1,000 ms, no conduction block is shown. At PCL of 370 ms, unidirectional conduction block occurs under the R858H condition. When the PCL is decreased, unidirectional conduction block can occur under other conditions. The maximum PCL (MPCL) that produced unidirectional conduction block for G1783C, wild-type (WT), P381S, M456I, A582D, and H858R is 350, 352, 354, 356, 365, and 370 ms, respectively.

To identify the electrophysiological substrates that promote arrhythmogenesis, tissue vulnerability to unidirectional conduction block necessary to the genesis of reentry was investigated, and space-time plots of AP propagation in the 1D transmural cable at different PCLs are shown in Figure 10. As can be seen, the tissue susceptibility to unidirectional conduction block showed a RT dependency and increased with a decrease in PCL. For instance, the MPCL (listed in Table S6) increased from 350 ms (G1783C) to 352 ms (WT), 354 ms (P381S), 356 ms (M456I), 365 ms (A582D), and 370 ms (R858H), respectively, with RT prolongation from 360.48 ms (G1783C) to 363.6 ms (WT), 367.34 ms (P381S), 374.84 ms (M456I), 381.14 ms (A582D), and 393.32 ms (R858H), respectively. As for R858H conditions, no unidirectional conduction block was observed at PCL = 1,000 ms, while unidirectional conduction block occurred at PCL = 370 ms. Bidirectional conduction was obtained in G1783C, WT, P381S, M456I, and A582D conditions at PCL = 370 ms. Therefore, R858H tissue was more vulnerable to unidirectional conduction block than other type tissues.

### Effects of *CACNA1C* mutations on dynamic behavior of reentrant excitation waves in 2D and 3D models

**Figure 11:**
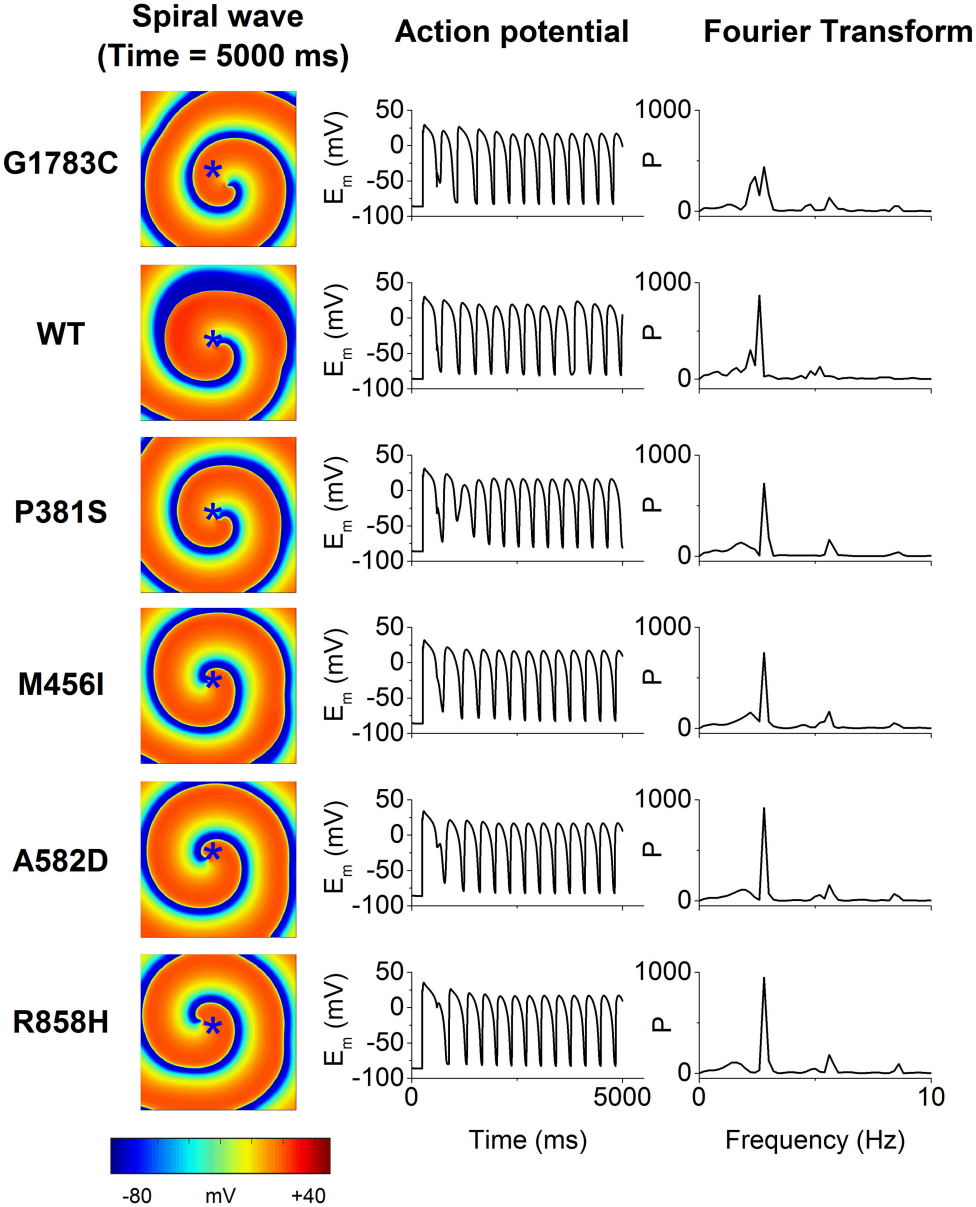
The spiral wave (Time = 5,000 ms, **left column**), action potential (E_m_, **middle column**) and temporal Fourier transforms of E_m_ (**right column**) for the G1783C, wild-type (WT), P381S, M456I, A582D, and R858H conditions. Action potential recorded from a representative point (*x* = 187.5 mm, *y* = 187.5 mm) that is marked by an asterisk.

To examine if mutations-induced changes in APDR curves promote the breakup of a spiral wave, an idealized homogeneous tissue model was constructed and spiral waves were induced by S1-S2 stimulation. Figure 11 shows snapshots of spiral waves (Time = 5,000 ms, left column), time series of action potentials (middle column) from the point indicated by an asterisk (left column) and power spectra (right column) for the G1783C, WT, P381S, M456I, A582D, and R858H conditions. Reentrant waves were stable and persistent. The fundamental frequencies obtained from these power spectra of action potentials were 2.8 Hz. These ordered plots of action potential provided evidence for the rotating spiral state.

**Figure 12:**
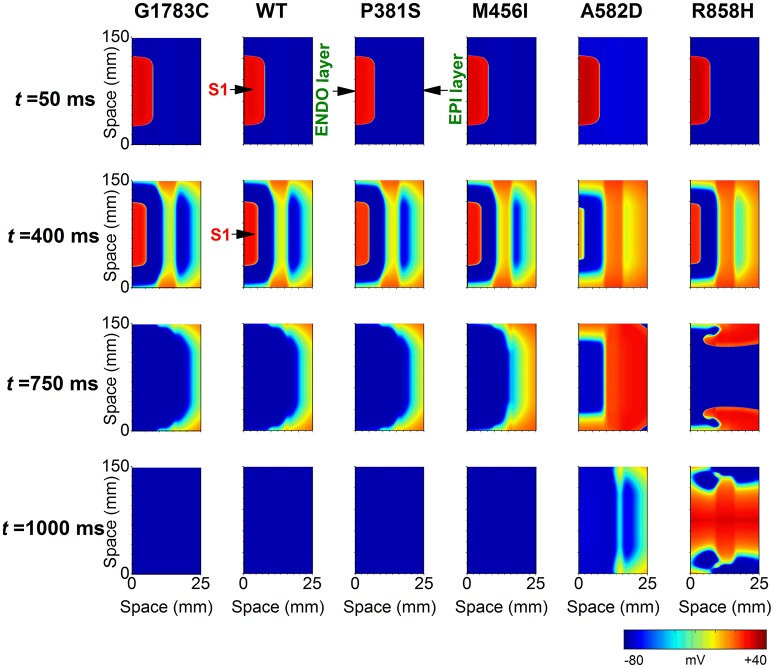
Snapshots of transmural conduction of electrical waves in a 24.75 × 150 mm^2^ transmual ventricular sheet. An electrical wave was elicited by the S1 stimulus applied to a 0.45 × 75 mm^2^ endocardial (ENDO) region at *t* = 0 ms and propagated from the ENDO layer to the epicardial (EPI) layer. Snapshot taken at *t* = 50 ms. Another S1 stimulus applied to the same ENDO region at *t* = 370 ms generated an electrical wave. Snapshot taken at *t* = 400 ms. Action potential propagation under G1783C, wild-type (WT), P381S, M456I, A582D, and R858H conditions, respectively. A spiral wave developed under the R858H condition at *t* = 750 ms. The pacing cycle length (PCL) used in these simulations is 370 ms.

To determine if R858H-induced changes were necessary to induce ventricular arrhythmias, an idealized transmural tissue model was developed to investigate the initiation of reentry with a S1-S1 stimulation. As is shown in Figure 12, when the PCL was 370 ms, the first S1 stimulus (*t* = 0 ms) produced a wave to propagate from the ENDO layer to the EPI layer. The second S1 stimulus (*t* = 370 ms) produced a conditioning wave and no unidirectional conduction block occurred under G1783C, WT, P381S, M456I, and A582D conditions (*t* = 750 ms). A unidirectional conduction block was initiated under the R858H condition, leading to the genesis of spiral waves (*t* = 750 ms) and fibrillation-like activity. The reentry wave for the R858H conditions was unstable and promoted self-termination when it collided with its own prolonged repolarization tail or tissue borders (*t* = 1,000 ms). These results support the previous notion that the MPCL of 2:1 block for the R858H mutation is 370 ms (shown in Figure 10) and unidirectional conduction block is responsible for the genesis of reentry. These 2D results concur with the 1D simulations data, further illustrating the pro-arrhythmic effects of the R858H mutation. In addition, reentry can be induced under other conditions (Figure S2) if the S1-S1 interval was decreased. For instance, the S1-S1 interval for initiating spiral waves was 365 ms (R858H), 340 ms (A582D), 335 ms (M456I), 320 ms (P381S), 315 ms (WT), and 310 ms (G1783C), respectively.

**Figure 13:**
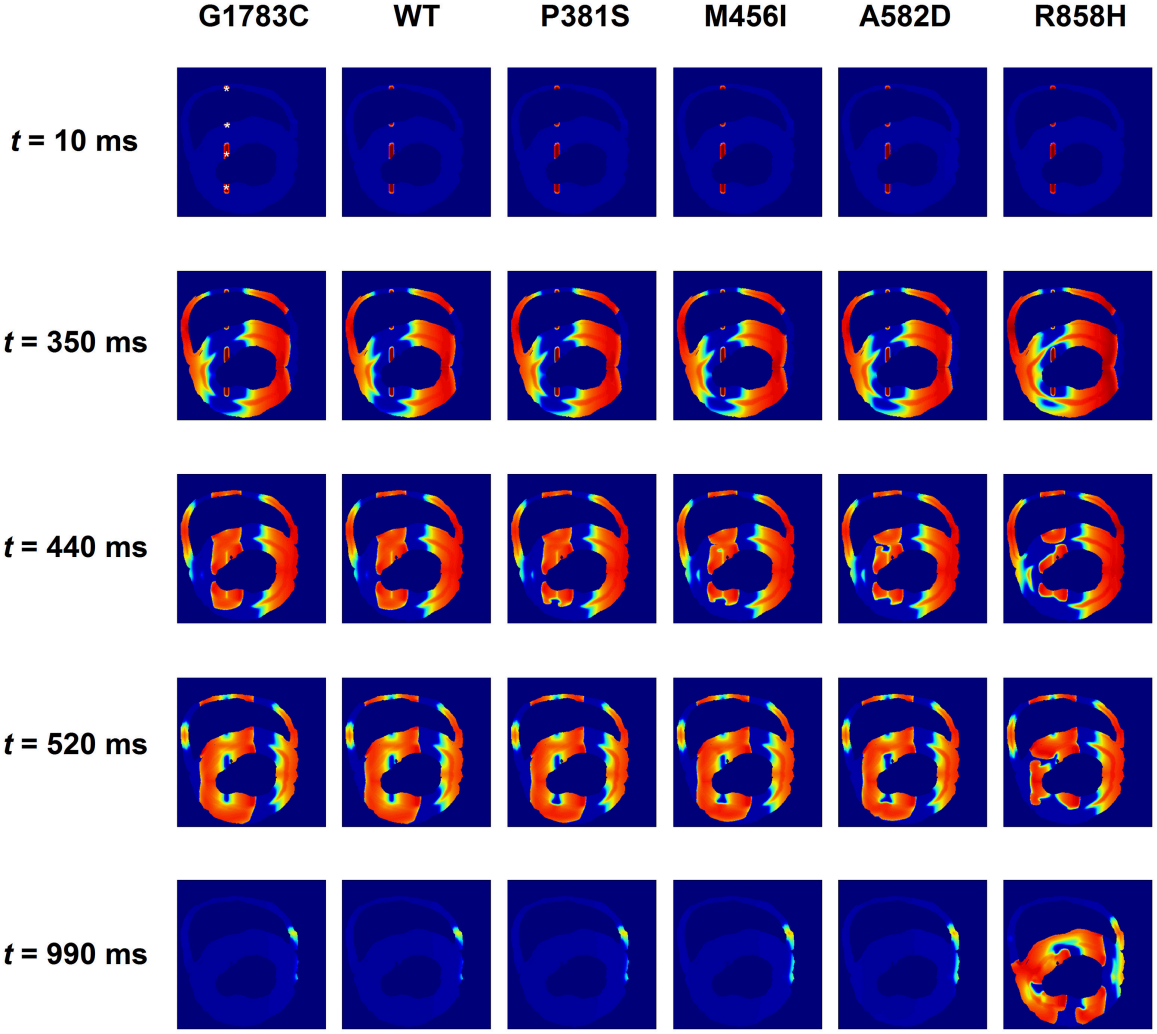
Snapshots of transmural conduction of electrical waves in a transmural ventricular slice. Electrical waves were elicited by the S1 stimulus applied to four endocardial (ENDO) sites (marked with white asterisks). Snapshot taken at *t* = 10 ms. Another S1 stimulus applied to the same sites at *t* = 350 ms generated several electrical waves. Action potential propagation under G1783C and wild-type (WT) conditions, local conduction block under P381S, M456I, and A582D conditions, and unidirectional conduction block under the R858H condition. Snapshot taken at *t* = 440 ms. Several spiral waves developed at *t* = 520 ms and sustained excitation waves at *t* = 990 ms under the R858H condition.

Further simulations were performed in a 2D slice model. Figure 13 shows that the R858H *I*_*CaL*_ induced reentrant spiral waves, which lead to sustained multiple reentrant wavelets in a 2D ventricular slice. Snapshots of excitation waves at different time points (*t* = 10, 350, 440, 520, and 990 ms) are shown for the G1783C, WT, P381S, M456I, A582D, and R858H conditions. As can be seen, a S1 stimulus was applied to the four pacing sites on the ENDO layer (marked with white asterisks, *t* = 10 ms) and excitation waves were initiated. At *t* = 350 ms, another S1 stimulus was used to induce spiral waves. At *t* = 440 ms, excitation waves propagated to the whole sheet under the G1783C and WT conditions, local conduction block was observed under the P381S, M456I, and A582D conditions, and unidirectional conduction block occurred under the R858H condition. Several spiral waves were produced at *t* = 520 ms and sustained reentry was observed at *t* = 990 ms. It was found that spiral waves can be induced under other conditions by decreasing S1-S1 interval. The S1-S1 interval for initiating spiral waves was 310 ms (G1783C), 315 ms (WT), 320 ms (P381S), 325 ms (M456I), 330 ms (A582D), and 345 ms (R858H), respectively (Figure S3). Detailed movies of spiral wave initiation can be found in Videos S1–S6.

**Figure 14:**
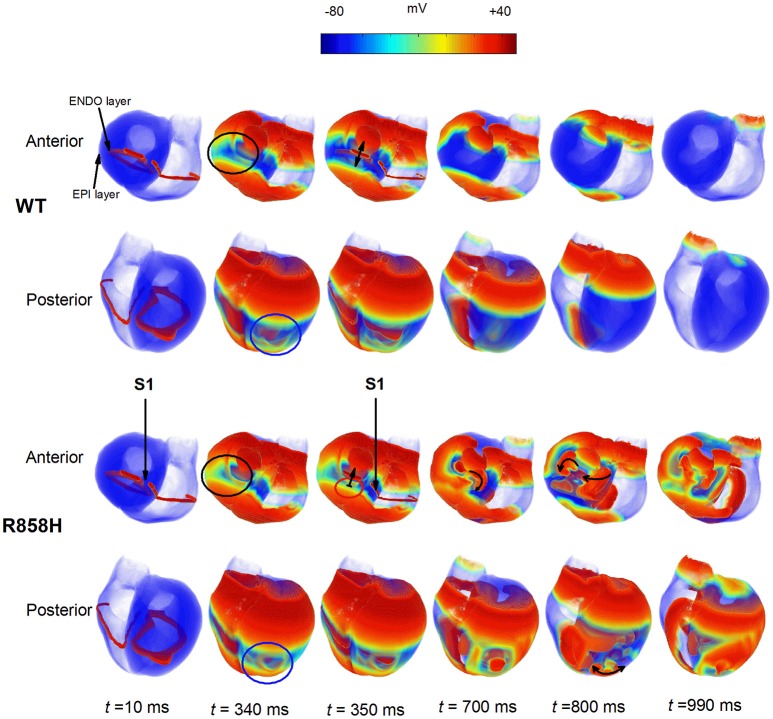
Dynamics of electrical waves in 3D human ventricles under wild-type (WT) and R858H conditions. The S1-S1 stimulation was applied to a 2.5 mm wide region of the endocardial (ENDO) layer to investigate the induction of reentry. A conditioning wave was initiated by the S1 stimulus. Snapshots taken at 10 ms. Compared with the R858H condition, the ventricles began to repolarize earlier under the WT condition at 340 ms (marked with a blue circle for the posterior view and a black circle for the anterior view). Another excitation wave initiated by the second S1 stimulus conducted bidirectionally (marked with a bidirectional arrow) under the WT condition whilst local conduction block (marked with unidirectional arrows) occurred (*t* = 350 ms) and spiral waves (marked with unidirectional arrows) were induced under the R858H condition (*t* = 700 ms and *t* = 800 ms). Snapshots for the anterior and posterior views were given. Unrecovered tissues were marked with a red circle and the S1-S1 interval was 348 ms.

To examine if R858H-induced changes promote ventricular arrhythmias in human ventricles, further simulations were performed using a 3D human heart geometry. As is shown in Figure 14, the first S1 stimulus (*t* = 10 ms) produced a wave to propagate from the ENDO layer to the EPI layer. Ventricular repolarization (marked with a black circle or a blue circle) under the WT condition occurred much earlier than that for the R858H settings (*t* = 340 ms). At *t* = 350 ms, another excitation wave initiated by the second S1 stimulus conducted bidirectionally (marked with a bidirectional arrow) under the WT condition whilst the wave was locally blocked (marked with unidirectional arrows) by unrecovered tissues (marked with a red circle). Therefore, spiral waves (marked with unidirectional arrows) were induced under the R858H condition (*t* = 700 ms and *t* = 800 ms), and re-entrant waves were persistent throughout the simulation (*t* = 990 ms). Moreover, when the S1-S1 interval was gradually decreased, spiral waves can also be induced (Figure S4). In details, the S1-S1 interval for initiating spiral waves was 250 ms (G1783C), 300 ms (WT), 309 ms (P381S), 340 ms (M456I), 345 ms (A582D), and 348 ms (R858H), respectively. Detailed movies of the spiral wave initiation can be found in Videos S7–S12.

Taken together, these data demonstrated that the R858H mutation facilitates initiation of reentrant excitation waves and suggested that the R858H tissue was more vulnerable to the initiation of reentrant excitation waves than other type tissues. A summary of the effects of *CACNA1C* mutated *I*_*CaL*_ on simulated human ventricular electrical activity is listed in Table 1.

**Table 1:** Summary of the effects of *CACNA1C* mutations (G1783C, WT, P381S, M456I, A582D, and R858H) on human ventricular electrical activity.

**Model**	**Quantity**	**G1783C**	**WT**	**P381S**	**M456I**	**A582D**	**R858H**
Subcellular (ENDO)	*I_*CaL*_* density (%)	86	100	104	108	119	154
	*[Ca^*2*+^]_*SR*(*m*)_* (mM)	2.54	2.68	2.82	2.94	3.17	3.51
	*[Ca^*2*+^]_*i*(*m*)_* (mM)	3.6e–4	4.0e–4	4.5e–4	4.9e–4	5.7e–4	7.0e–4
Cell (ENDO)	Resting potential (mV)	−85.87	−85.83	−85.80	−85.75	−85.67	−85.58
	APD (ms)	264.4	267.8	271.8	277	282.6	294
	APDR maximal slope	1.02	1.08	1.1	1.15	1.15	1.35
1D	CV (mm/ms)	0.344	0.344	0.344	0.344	0.344	0.344
	MPCL (ms)	350	352	354	356	365	370
	T wave width (ms)	60	59.9	59.9	61.7	63.9	64.5
	QT (ms)	397.1	398.5	401.2	408.2	415.9	425.7
2D MCELL tissue	Life span (ms)	>5,000	>5,000	>5,000	>5,000	>5,000	>5,000
	Dominant frequency (Hz)	2.8	2.8	2.8	2.8	2.8	2.8
2D transmural tissue (S1-S1 = 365 ms)	Life span (ms)	<1,000	<1,000	<1,000	<1,000	<1,000	>1,000
	S1-S1 interval (ms)	310	315	320	335	340	365
2D slice tissue (S1-S1 = 345 ms)	Life span (ms)	<1,000	<1,000	<1,000	<1,000	<1,000	>1,000
	S1-S1 interval (ms)	310	315	320	325	330	345
3D model (S1-S1 = 348 ms)	Life span (ms)	−	<1,000	–	–	–	>1,000
	S1-S1 interval (ms)	250	300	309	340	345	348

## Discussion

### Summary of major findings

To our knowledge, this is the first study to investigate mechanisms underlying the genesis of VF in patients with the *CACNA1C* R858H mutation. The study found five major findings, (i) due to the R858H mutation *I*_*CaL*_ caused intracellular calcium overload, resulting in afterdepolarizations in single cells and focal activity in 1D fiber tissues; (ii) The R858H mutation induced an increase in *I*_*CaL*_ prolonged APD and augmented RT, leading to QT interval prolongation; (iii) The R858H mutation-induced electrical differences between cells augmented electrical heterogeneity, causing repolarization dispersion and thereby increasing T-wave width; (iv) Although, the R858H mutation steepened the APDR relationships in single cells, a stable spiral wave remained in homogeneous tissues; (v) Changes in cellular electrophysiology modulated wave conduction at tissue level facilitating unidirectional conduction block and thereby increasing tissue susceptibility to VF genesis in transmural ventricular tissues.

These simulation data in this study constitute novel evidence that the pro-arrhythmic effects of *I*_*CaL*_ associated with Ca_V_1.2 R858H mutation involve both increased cell susceptibility to afterdepolarizations and tissue vulnerability to the reentry. The effects of the R858H mutation were investigated at cellular, 1D strand and 2D tissue and 3D organ levels, showing not only alterations in calcium handling and QT prolongation, but also repolarization dispersion and reentry.

### Computer modeling of *CACNA1C* mutations

In this study, the electrophysiological consequences of *CACNA1C* mutations were investigated by modeling the *I*_*CaL*_, as in previous studies (Faber et al., 2007; Zhu and Clancy, 2007; Sung et al., 2010; Morotti et al., 2012; Boczek et al., 2015a; Bai et al., 2016c). Based on experimental data on *I*_*CaL*_ current densities, steady-state activation curves, voltage-dependent inactivation curves as well as the time constant for the voltage-dependent inactivation (Fukuyama et al., 2014), the *I*_*CaL*_ model was developed by changing the *I*_*CaL*_ conductance, the half-activation voltage, the slope of the activation curve, the half-inactivation voltage, the slope of the inactivation curve and the scaling factor of inactivation time constant. The “mutant” *I*_*CaL*_ models successfully reproduced the I-V relationships obtained from experimental studies (Fukuyama et al., 2014). These *I*_*CaL*_ models were incorporated into a human ventricular cell model, allowing us to relate changes in *I*_*CaL*_ to AP at the cellular level, in agreement with previous studies (Splawski et al., 2005; Sung et al., 2010; Wemhöner et al., 2015; Bai et al., 2016c). According to transmural ventricular wedge preparation models (Gima and Rudy, 2002; O'Hara et al., 2011), the 1D transmural strand model, integrating human ENDO-, MCELL- and EPI- cells, was constructed to compute unipolar pseudo-ECGs. In the cable, the proportion of each region composed of each distinct cell type is consistent with that used in other studies (O'Hara et al., 2011). In agreement with clinical findings, R858H-induced *I*_*CaL*_ led to a QT interval of 425.7 ms, which is within the QT range (420–476 ms) of R858H-porbands (Fukuyama et al., 2014). These models could be considered as a first step toward the validation of electrophysiological model. Thus, cardiac models provide a powerful tool for the study of mechanisms underlying ventricular arrhythmias caused by *CACNA1C* mutation effects.

Previous studies have demonstrated that distinct mutations can have variable effects on current morphology and lead to varying degrees of electrophysiological consequences, depending on kinetic changes induced by the *CACNA1C* mutation (Sung et al., 2010; Wemhöner et al., 2015; Sutphin et al., 2016). The “mutant” *I*_*CaL*_ represents state-specific kinetic properties of ion channels, allowing us to relate functional changes in *I*_*CaL*_ to AP at the cellular level, the electrocardiogram characteristics at the fiber tissue level and the spatiotemporal behavior of excitation waves at the sheet tissue level. In the present study, although G1783C, P381S, M456I, A582D, and R858H mutations changed Ca_V_1.2 in activation and inactivation, VF was only observed in R858H patients (Fukuyama et al., 2013, 2014). Therefore, we have focused on the R858H mutation marked by an increase in the *I*_*CaL*_ and have investigated mechanisms underlying the genesis of reentry.

### Ionic mechanism of afterdepolarizations in R858H cells

These analyses of R858H revealed an unexpected, distinct electrophysiological phenotype from the classical TS mutations (G406R/G402S; Splawski et al., 2004, 2005). Electrophysiological studies of G406R in exon 8A and G406R/G402S in exon8 showed almost complete loss of inactivation of Ca_V_1.2 (Splawski et al., 2004, 2005). In contrast, R858H showed a significant gain of current density, ~2 mV negative shift of activation and ~2 mV positive shift of inactivation, resulting in an increased window current (Fukuyama et al., 2014). Due to the different electrophysiological phenotypes between the canonical G406R/G402S mutations and R858H, we performed modeling studies to better understand how the increased window current may affect the ventricular AP. Like the modeling studies of G406R/G402S (Splawski et al., 2004; Zhu and Clancy, 2007), our results showed prolongation of APD in TP06 and O'Hara-Rudy dynamic (ORd) human ventricular cell models (Figure S5). At fast pacing rates, R858H-induced SR calcium loading, which may lead to spontaneous afterdepolarizations, was also observed (Figure S6). The result of R858H-induced afterdepolarizations is consistent with other reports that pro-arrhythmic actions of *CACNA1C* mutations are due to SR calcium leak, thereby enabling an *I*_*NCX*_ that triggers afterdepolarizations (Splawski et al., 2005). In agreement with experimental findings (Priori and Corr, 1990), the R858H induced EADs and DADs share the same mechanism, where NCX-mediated inward current is involved.

These afterdepolarizations may mainly result from an increase *I*_*CaL*_ arising from the R858H mutation, because the VF in the R858H patients was completely prevented by atenolol, a beta-blocker (Fukuyama et al., 2014). Thus, our results support the concept that the R858H-induced defect is sufficient to cause significant action potential prolongation and afterdepolarizations in ventricular myocytes. Our data highlight how small changes in cellular calcium entry through Ca_V_1.2 can lead to far-reaching changes in action potentials and intracellular calcium handling.

Computational modeling has shed valuable light on the cellular mechanisms underlying ventricular arrhythmia. Splawski et al. showed that a G406R mutation prolonged action potentials, altered intracellular calcium handling, and triggered DADs (Splawski et al., 2005). Sung et al. showed that G406R-induced EADs and DADs shared the same mechanism, with NCX-mediated inward current (Sung et al., 2010). However, how the G406R mutation is linked to ventricular arrhythmias is not yet fully understood. Thiel et al. showed that a G406R mutation altered intracellular calcium handling and calcium signaling, which could in turn contribute to DADs due to calcium signaling-induced SR calcium overload (Thiel et al., 2008). Recently, Dick et al. suggested that TS mutations (G406R/ G402S) disrupted calcium-dependent inactivation, which played an important role in APD prolongation and development of EADs (Dick et al., 2016). Different from the mechanism underlying G406R-induced arrhythmias, an I1166T mutation led to an EAD due to *I*_*CaL*_ reactivation (Boczek et al., 2015a). Other *CACNA1C* mutations also caused QT prolongation and cardiac arrhythmias (Gillis et al., 2012; Boczek et al., 2013, 2015b; Fukuyama et al., 2013, 2014; Hennessey et al., 2014; Wemhöner et al., 2015; Landstrom et al., 2016; Sutphin et al., 2016), but their cellular mechanisms underlying cardiac arrhythmias remain relatively unexplored, and the causal link between the *CACNA1C* mutations and ventricular arrhythmias is not addressed directly. In this study, the cellular mechanism that *I*_*CaL*_ due to the R858H mutation prolongs APD and increases SR calcium load, resulting in spontaneous *I*_*rel*_ and afterdepolarizations, is proposed. These simulation data of the present study add to the growing weight of evidence implicating cellular mechanisms of *CACNA1C* mutations.

### Reentry initiation mechanism

The LQT8 is associated with malignant ventricular arrhythmias and a patient with a R858H mutation experienced an episode of VF (Fukuyama et al., 2013, 2014). Our data showed that R858H-induced electrical heterogeneity increased the transmural DOR and favored unidirectional conduction block necessary to the development of VF (Zhu and Clancy, 2007; Sung et al., 2010). The simulations may provide evidence for the pro-arrhythmic effects of the R858H mutation in facilitating reentrant excitation waves. They support the notion that DOR is a marker of tissue electrical heterogeneity and a substrate for reentrant arrhythmias (Antzelevitch and Fish, 2001; Chauhan et al., 2006). In agreement with previous studies (Zhang et al., 2008), cell simulation results indicate that membrane potential differences and inhomogeneous APDs of ventricular cardiomyocytes contribute to the tissue electrical heterogeneity. As for membrane potential differences, afterdepolarizations became inducible under the R858H condition when the SR calcium load was progressively increased in the MCELL cells. The maximal membrane potential differences both ENDO-M and EPI-M APs were significantly increased. As for inhomogeneous APD, the R858H mutation produced inhomogeneous APD prolongation in EPI, MCELL and ENDO cells, leading to the lager spatial gradient of APD along the transmural ventricular wall. Therefore, these electrical differences due to the R858H mutation at the cellular level contributed to an augmented transmural DOR, increasing tissue vulnerability to unidirectional conduction block.

Linking APD prolongation to unidirectional conduction block can provide insights into the risk of cardiac arrhythmias. The APD corresponding to the R858H model was longer than that of other models at S1-S1 = 1000 ms. The RT prolongation was consistent with the prediction of APD. At S1-S1 = 370 ms, the AP maps showed 2:1 block under the R858H condition. A similar 2:1 block was also observed under rapid conditions in the other heart models. Moreover, in the R858H heart, a local conduction block corresponded to unrecovered tissues and spiral waves were induced (Figure 14, the R858H heart model at S1-S1 = 348 ms). As the S1-S1 interval was further decreased (at PCL = 300 ms), we observed unidirectional conduction block and reentrant waves in the WT ventricles (Figure S4). This suggested that the prolonged APD tissues formed excitable obstacles which can anchor spiral waves.

Our simulations indicate that the pro-arrhythmic effect of the R858H mutation is reflected by the increased MPCL of 2:1 block. This is compatible with observations from previous studies (Zhu and Clancy, 2007), in which a long PCL led to 2:1 block under the G406R/G402S mutation condition. Compared with other mutations, unidirectional conduction block under the R858H condition occurred at a longer S1-S1 interval, implying higher risk of cardiac arrhythmias in R858H patients at relatively slow heart rates. Furthermore, the 2D and 3D simulated results support the notion that the R858H mutation increases the proarrhythmic risk. Although the spiral waves can be induced under all conditions, a relatively long S1-S1 interval is required for the initiation of reentrant waves under the R858H condition.

### Mechanisms for VF

VF is the most common cause of sudden cardiac death. Fibrillation results when an electrical wave break induces reentry and triggers a cascade of new wave breaks (Weiss et al., 2005). In our simulations, three potential mechanisms underlying VF caused by the R858H mutation were considered, (a) the restitution hypothesis; (b) afterdepolarizations-mediated fibrillation hypothesis; and (c) dispersion of refractoriness hypothesis.

According to the restitution hypothesis, the chaotic excitation dynamics during VF are the result of dynamical instabilities in APD, the occurrence of which requires that the slope of the APD restitution curve exceeds 1 (Fenton et al., 2002). Although the APDR relationships were steepened by the R858H mutation, stable spiral wave dynamics occurred in homogeneous ventricular tissues. The measured maximum slope of APDR for the EPI cell was 1.5 which didn't exceed the minimum APDR slope (1.5) for the occurrence of spiral breakup in the TP06 model (Ten Tusscher and Panfilov, 2006a). Also, a spiral wave remained in the MCELL tissue with the APDR slope of 2 which was within the range of experimentally measured slopes (1.28–3.78; Pak et al., 2004). In addition, the slopes measured in the ORd model were within 1 under the R858H condition (Figure S7). Thus, our study confirmed that the effects of R858H-induced *I*_*CaL*_ are model independent. These results indicated that steep APDR is not the mechanism of VF caused by the R858H mutation.

According to afterdepolarizations-mediated fibrillation hypothesis, abnormal electrical excitations caused by afterdepolarizations can disrupt the normal propagation of electrical waves and cause life-threatening arrhythmias like VF. The mechanisms of afterdepolarizations generation include the reactivation of *I*_*CaL*_ and the enhancement of *I*_*NCX*_ arising from spontaneous calcium release. Simulation studies showed that three types of spiral fibrillation emerged when EAD was mainly induced by reactivation of *I*_*CaL*_ (Vandersickel et al., 2014; Zimik et al., 2015). For the R858H condition, afterdepolarizations were caused by the enhancement of the *I*_*NCX*_ arising from spontaneous calcium release. Although, focal activity occurred in the 1D fiber tissue, electrical coupling between cells suppressed the formation of PVC under the R858H condition. Experimental and computational studies were done to investigate how clumps of cells, eliciting afterdepolarizations in synchrony, give rise to triggered activities, which can disturb any prevailing course of wave propagation and induce electrical-wave turbulence (Sato et al., 2009; de Lange et al., 2012; Myles et al., 2012). Local synchronization of afterdepolarizations cells was suggested the mechanism underlying the formation of PVCs. Of course, our previous study has demonstrated that afterdepolarizations induced by SR calcium overload are synchronized to overcome the source-sink mismatch and produce PVCs in cardiac tissues (Bai et al., 2017). These studies support the notion that R858H-induced afterdepolarizations in MCELL cells are implicated in the induction of electrical-wave turbulence.

According to the dispersion of refractoriness hypothesis, wave break is produced by electrophysiological and anatomic heterogeneities in the tissue (Xie et al., 2001). For the R858H condition, intrinsic electrophysiological heterogeneity was sufficiently increased by the altered *I*_*CaL*_ due to a R858H mutation. Electrophysiological heterogeneity produced DOR responsible for 2:1 conduction block necessary for the fibrillation-like activity. Simulation studies also demonstrated that this breakup mechanism did not require steep APDR (Xie et al., 2001; Fenton et al., 2002). Thus, these data indicated that DOR was a breakup mechanism for the R858H condition.

Taken together, steep APDR is not the mechanism responsible for VF in the current work. Our results suggest that R858H induced afterdepolarizations and transmural APD dispersion are responsible for the potential mechanisms underlying the formation of VF. For afterdepolarizations-mediated fibrillation, it must be synchronized across many cells (Xie et al., 2010; Myles et al., 2012). And DOR caused by the R858H mutation increased the susceptibility to dynamic instabilities.

### Clinical implications

The presented results showed a QT interval prolongation from 397.1 ms (WT) to 425.7 ms (R858H), which was within the range of the QTc interval (420–476 ms) in R858H-probands (Fukuyama et al., 2014). Thus, a bridge between *CACNA1C* mutations and pro-arrhythmic phenotypes was built by the cardiac model. Also, the model can be used to investigate the effect of therapies targeting selected ion channels. More importantly, defining a classification based on a reliable VF risk factor would be very useful in guiding the selection of LQT8 patients who are in need of a ventricular defibrillator. In the current work, we have shown that, DAD-mediated AP at fast pacing rates isn't shortened but further prolonged, indicating QT interval prolongation at fast pacing (Viskin et al., 2010; Adler et al., 2012; Kaye et al., 2013). The QT abnormality may become a clinical marker to determine the VF risk in LQT8 patients.

### Limitations of the study

The TP06 model was used to investigate the pro-arrhythmic effects of *CACNA1C* mutations and its limitations were discussed elsewhere (Zhang et al., 2008; Bai et al., 2016c). Here, we address several limitations in this study. (i) Due to the lack of a precise model of complex calcium cycling, this study does not consider the effect of a calcium spark on the genesis of afterdepolarizations and further refinement of the model is required. (ii) Our simulations show that R858H-induced defects can lead to the cellular arrhythmogenesis in the absence of neural influences. However, clinically observed arrhythmias typically occur in the setting of increased sympathetic tone (Best and Kamp, 2010), such as crying, and so future efforts will expand the study to investigate the effects of the autonomic nervous system on the development of cardiac arrhythmias. (iii) To maintain structures of the TP06 (Ten Tusscher and Panfilov, 2006a) and ORd (O'Hara et al., 2011) models, the original *I*_*CaL*_ conductance was used for the WT condition and the maximum conductance of each “mutant” *I*_*CaL*_ was determined by scaling relative current proportions. In agreement with the TP06 model (Ten Tusscher et al., 2004; Ten Tusscher and Panfilov, 2006a), the *I*_*CaL*_ was same for ENDO, MCELL and EPI cells in the ORd model (O'Hara et al., 2011) and electrophysiological heterogeneity caused by different *I*_*CaL*_ densities for three cell types was not considered in this study. Thus, special attention must be paid to explain these simulated data. (iv) In the multicellular tissue model, due to a lack of detailed experimental data on cell properties and well-delineated regions, the proportion of each region composed of each distinct cell type was chosen to produce a positive T-wave in the 1D transmural cable, similar to those used in other studies (Gima and Rudy, 2002; O'Hara et al., 2011). However, simulated ECG, which might be influenced by torso effects and the dimensions of the regions, wasn't completely consistent with clinical findings (Fukuyama et al., 2014). (v) Fibrillation at the tissue level resulted from afterdepolarizations at a single-cell level was not observed, but modeling and experimental studies have suggested that spatial-temporal synchronization of SR calcium overload and release can overcome the source-sink mismatch and produce focal arrhythmia in the heart (Xie et al., 2010; Myles et al., 2012), and so future efforts will expand the study to investigate that how the heart produces spatial-temporal synchronization of afterdepolarizations and the R858H mutation triggers VF in the heart. (vi) In the 1D and 2D tissue models, we assumed isotropic cell-to-cell electrical coupling. On the one hand, possible anisotropic intercellular electrical coupling may play important roles in the initiation and perpetuation of reentry. On the other hand, omitting anisotropy can be useful, in that changes to tissue behavior observed in the present study can be attributed with confidence to the implemented modifications to *I*_*CaL*_. (vii) The space step for the 2D homogeneous ventricular model is different from that used for 2D transmural ventricular sheet. The space step is chosen to produce a stable spiral wave close to those in other studies (Ten Tusscher and Panfilov, 2006a; Vandersickel et al., 2014; Nayak and Pandit, 2015; Zimik et al., 2015; Nayak et al., 2017), in that rotating spiral can be attributed with confidence to the APDR slope which is below the minimum value for the occurrence of electrical instability. (viii) Although the 2D homogeneous tissue was designed to examine if mutations-induced changes in APDR curves promote the breakup of a spiral wave and we expected the simulated results to hold in tissues of reasonable size, a reasonable domain cannot avoid the frequent termination of spiral waves by collisions with the boundaries of the simulation domain. Therefore, the size of the 2D homogeneous tissue chosen is similar to that used in other studies (Ten Tusscher and Panfilov, 2006a; Vandersickel et al., 2014; Nayak and Pandit, 2015; Zimik et al., 2015; Nayak et al., 2017), but it is larger than that of a typical human heart. Special attention must be paid to explain these simulated data. (ix) Due to a lack of fiber orientation data, fiber orientation was not included in the 3D model and its effect on reentry was not studied. Nevertheless, whilst it is important to make explicit the potential limitations of the approaches adopted in the present study, these potential limitations are not expected to influence fundamental conclusions that can be drawn from our data on the mechanisms by which increased *I*_*CaL*_ due to the R858H mutation can increase risk of ventricular arrhythmias.

## Conclusions

Computational modeling indicated that AP shape at the cellular level, ECG and unidirectional conduction block at the fiber tissue level, and spiral waves at the sheet tissue level as well as at the organ level under the R858H condition, provided a causal link between a R858H mutation and VF. Based on our findings, we propose that R858H induced transmural APD dispersion promotes the genesis of VF.

## Author contributions

JB, KW, and HZ conceived and designed the experiments; JB performed the simulations, prepared figures and analyzed the results; JB, KW, and HZ drafted and edited the manuscript. YLiu, GL, SD, YLi, CL, and YY gave valuable suggestions. All authors reviewed the final version of the manuscript.

### Conflict of interest statement

The authors declare that the research was conducted in the absence of any commercial or financial relationships that could be construed as a potential conflict of interest.

## Abbreviations

VF: Ventricular fibrillation
PVC: premature ventricular complex
LQTS: long QT syndrome
LQT8: LQTS type 8
TS: timothy syndrome
WT: wild-type
ECC: excitation-contraction coupling
1D, 2D, and 3D: one-two- and three- dimensional
*I*_*CaL*_: L-type calcium current
*I*_*NCX*_: sodium-calcium exchanger current
*I*_*rel*_: the calcium-induced-calcium release flux
*I*_*rel*(*m*)_: *I*_*rel*_ amplitude
*I*_*to*_: the transient outward potassium channel current
*I*_*Ks*_: slow delayed rectifier potassium channel current
*C*_*m*_: the capacitance
*D*: the effective diffusion constant
*I*_*ion*_: the total transmembrane current
*V*_*m*_: the trans-membrane potential
*[Ca*^*2*+^*]*_*i*_: cytoplasmic calcium concentration
*[Ca*^*2*+^*]*_*i*(*m*)_: *[Ca*^*2*+^*]*_*i*_ amplitude
SR: sarcoplasmic reticulum
*[Ca*^*2*+^*]*_*SR*_: SR calcium concentration
*[Ca*^*2*+^*]*_*SR*(*m*)_: SR calcium content
AP: action potential
APD: AP duration
APD_90_: APD at 90% repolarization
APDR: APD restitution
ENDO: endocardial-cell
MCELL: midmyocardial-cell
EPI: epicardial-cell
ENDO-EPI: between endocardial- and epicardial- cells
ENDO-M: between endocardial- and midmyocardial- cells
EPI-M: between epicardial- and midmyocardial- cells
EAD: early afterdepolarization
DAD: delayed afterdepolarization
TP06 model: Ten Tusscher-Panfilov human ventricular cell model
ORd model: O'Hara-Rudy dynamic human ventricular cell model
I-V relationships: current-voltage relationships
*CSF*: the scaling factor of the *I*_*CaL*_ conductance
*V*_*a, 0.5*_: the midpoint voltage of the voltage-activation curve
*S*_*a*_: the slope of the voltage-activation curve
*V*_*ina, 0.5*_: the midpoint voltage of the voltage-inactivation curve
*S*_*ina*_: the slope of the voltage-inactivation curve
*TCSF*: the scaling factor of voltage-inactivation time constant
PCL: pacing cycle length
MPCL: the maximum PCL that produced 2:1 block
DOR: dispersion of repolarization
RT: repolarization time
SG: spatial gradient of APD
MSG: maximal SG
δ: the membrane potential gradient
CV: conduction velocity
DI: diastolic interval.
