# Supplementary figures and images for: Computational Cardiac Modeling Reveals Mechanisms of Ventricular Arrhythmogenesis in Long QT Syndrome Type 8: CACNA1C R858H Mutation Linked to Ventricular Fibrillation
Source: Front Physiol. 2017 Oct 4;8:771. doi: 10.3389/fphys.2017.00771 (PMC5632762; doi:10.3389/fphys.2017.00771)

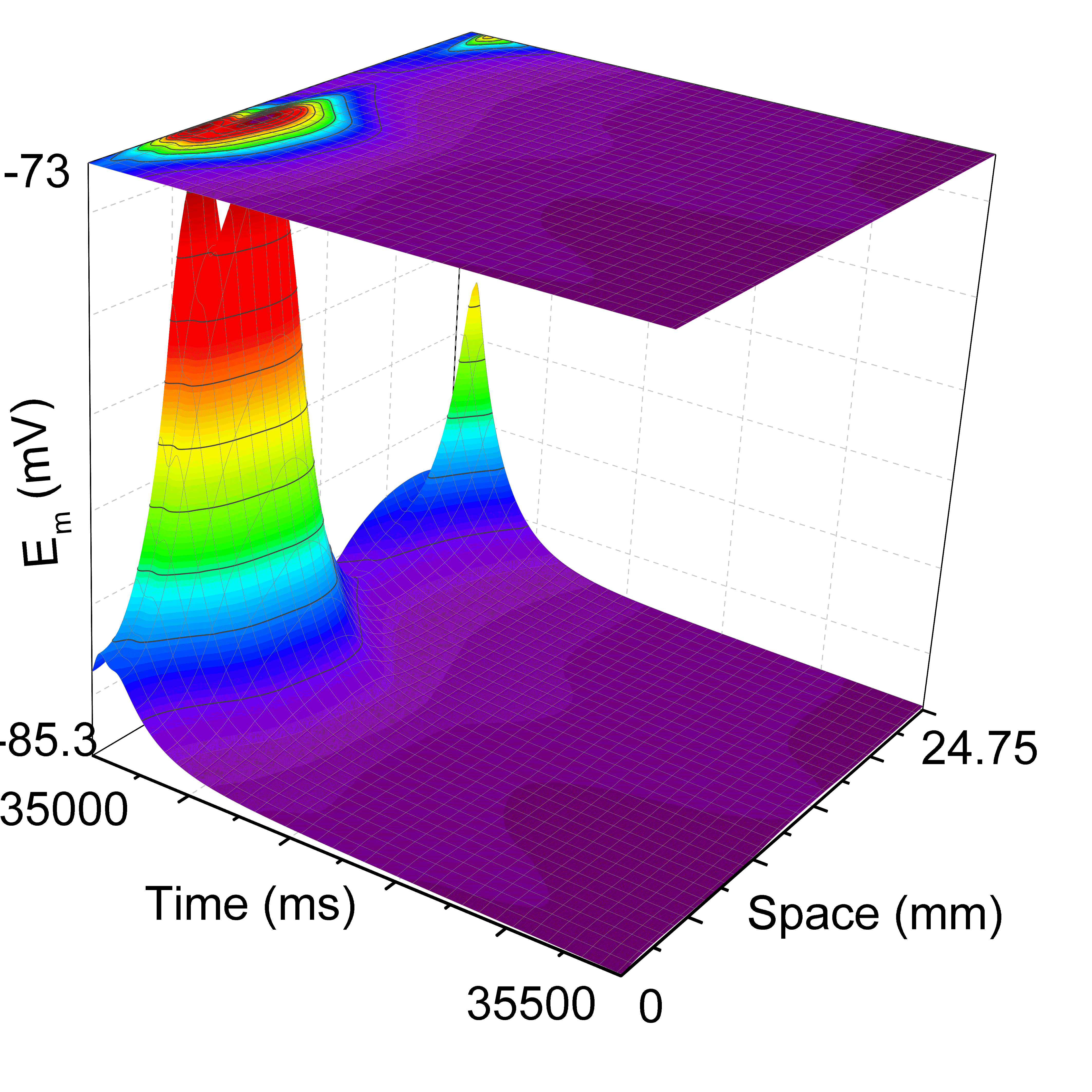

Supplement: Supplementary file 1 [file Image1.TIF]

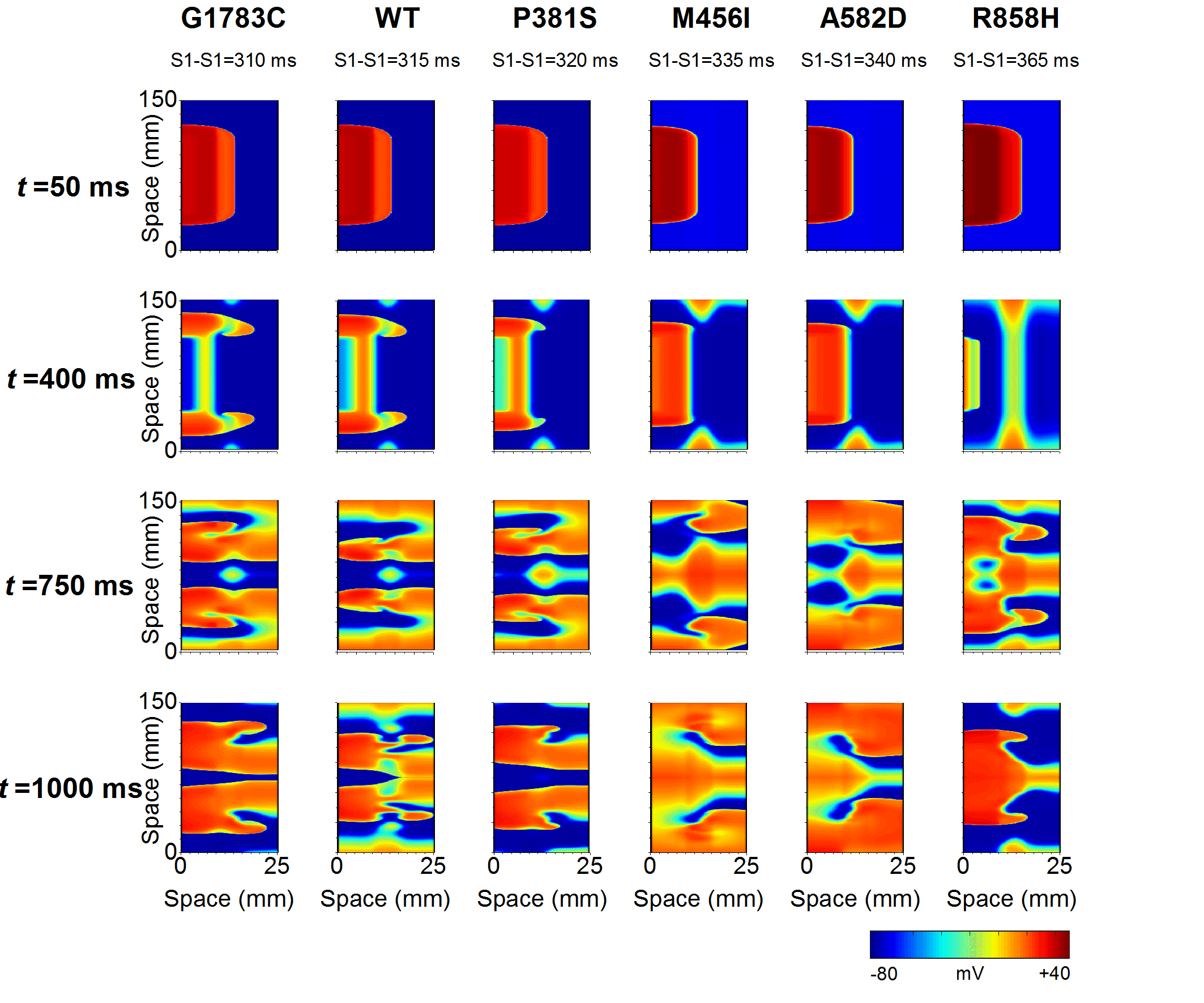

Supplement: Supplementary file 2 [file Image2.TIF]

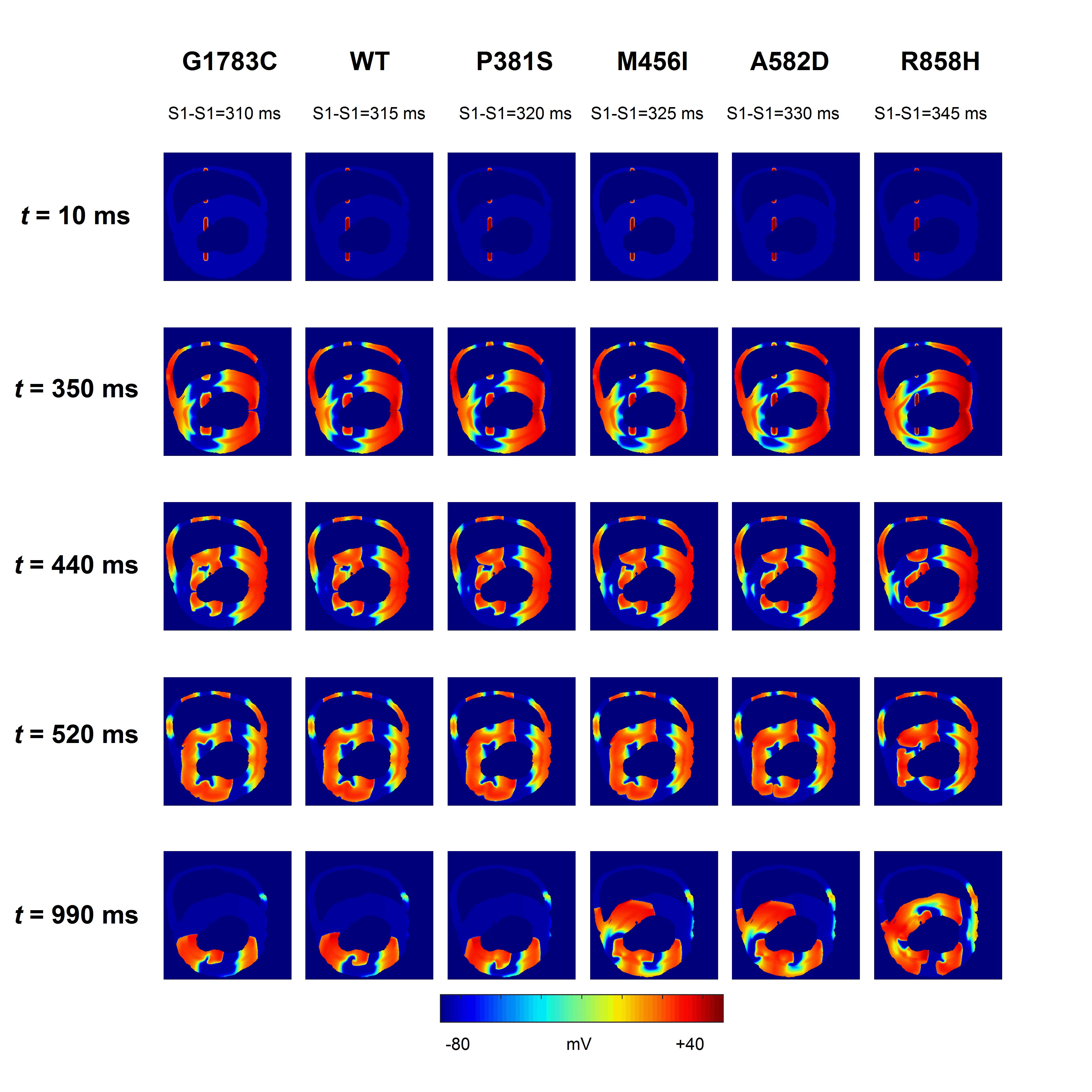

Supplement: Supplementary file 3 [file Image3.TIF]

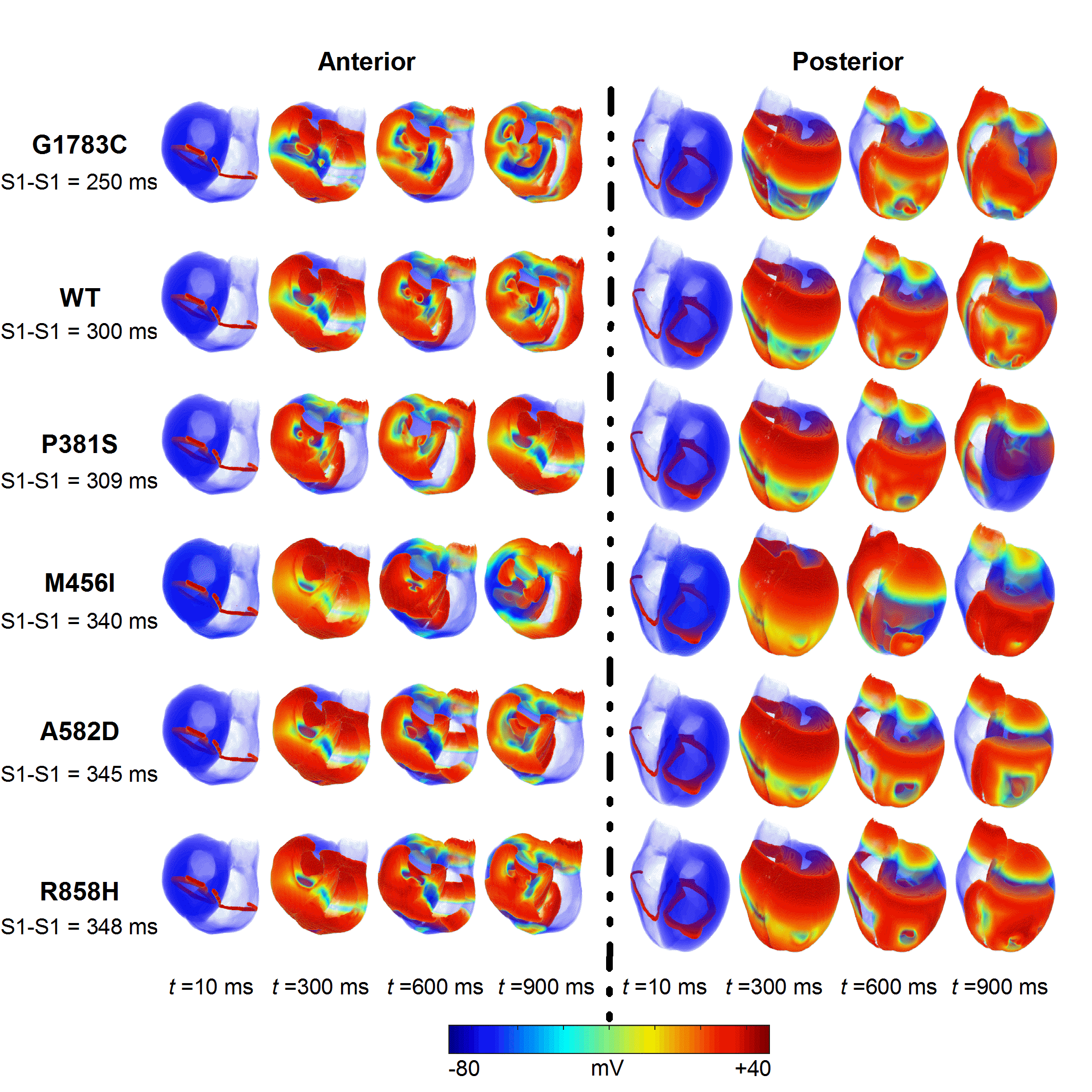

Supplement: Supplementary file 4 [file Image4.TIF]

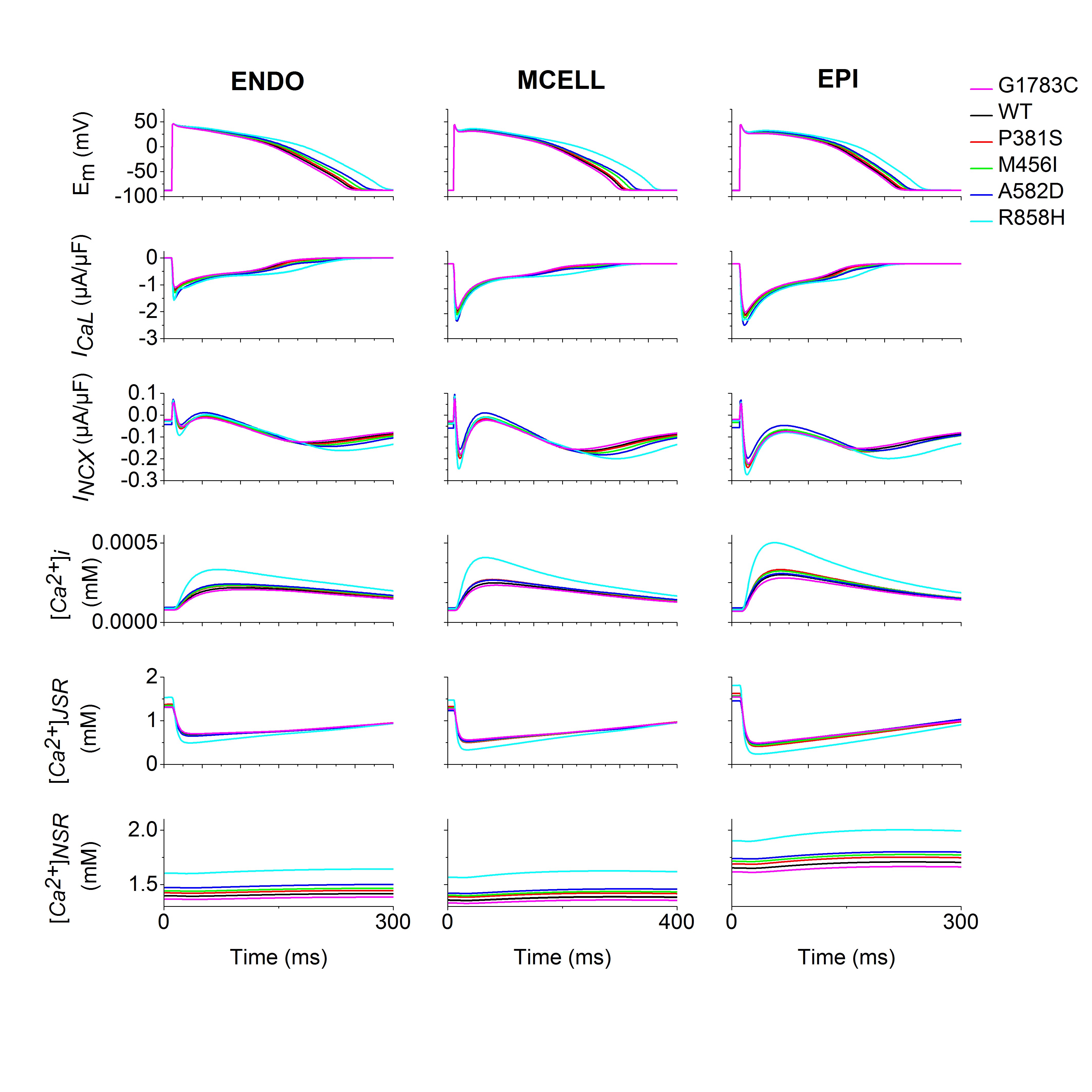

Supplement: Supplementary file 5 [file Image5.TIF]

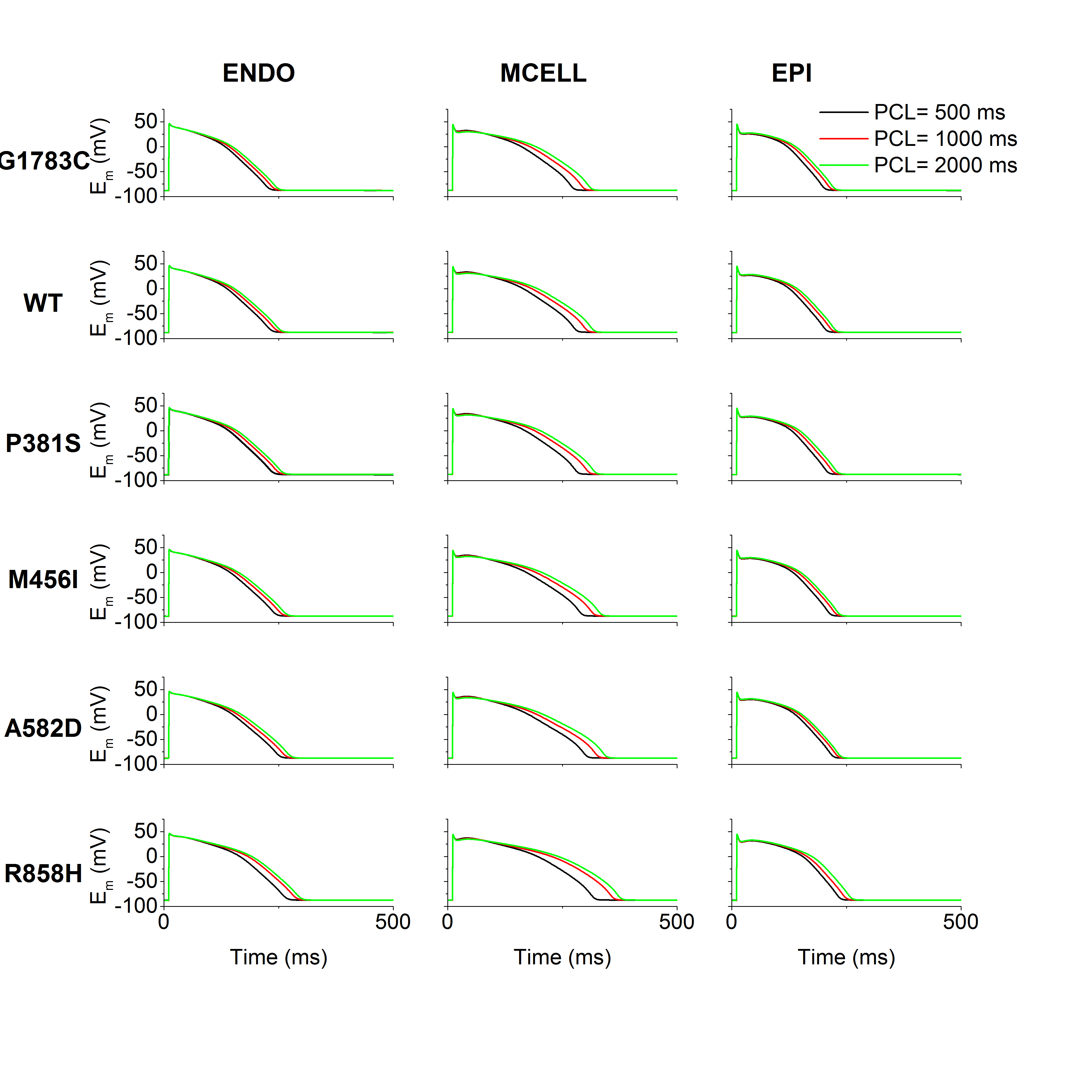

Supplement: Supplementary file 6 [file Image6.TIF]

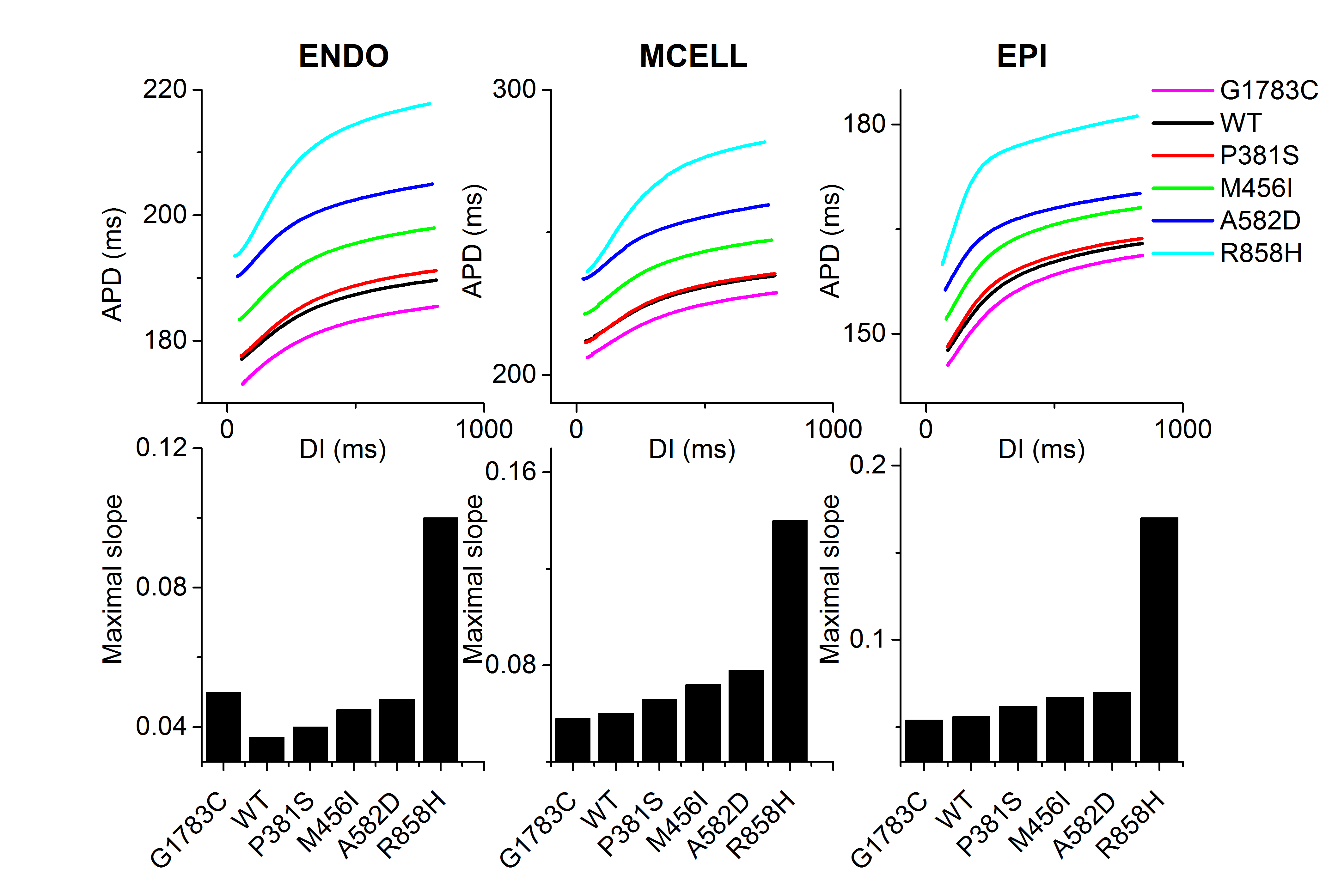

Supplement: Supplementary file 7 [file Image7.TIF]
