# Supplementary material for: Computational Cardiac Modeling Reveals Mechanisms of Ventricular Arrhythmogenesis in Long QT Syndrome Type 8: CACNA1C R858H Mutation Linked to Ventricular Fibrillation
Source: Front Physiol. 2017 Oct 4;8:771. doi: 10.3389/fphys.2017.00771 (PMC5632762; doi:10.3389/fphys.2017.00771)
Supplement: Supplementary file 20 [file DataSheet1.DOCX]

Supplementary Material

Computational cardiac modelling reveals [mechanisms of ventricular](http://scholar.google.co.jp/scholar?q=reveals+mechanism+of+ventricular+arrhythmias&hl=zh-CN&as_sdt=0&as_vis=1&oi=scholart&sa=X&ved=0ahUKEwiJ2Magps3SAhXFwLwKHf3jAoQQgQMIGzAA) arrhythmogenesis in long QT syndrome type 8: *CACNA1C* R858H mutation linked to ventricular fibrillation

Jieyun Bai^*^, Kuanquan Wang^*^, Yashu Liu, Yacong Li, Cuiping Liang, Gongning Luo, Suyu Dong, Yongfeng Yuan, Henggui Zhang^*^

*** Correspondence:** Jieyun Bai: Jieyunbai@hit.edu.cn

Kuanquan Wang: wangkq@hit.edu.cn, [wangkq@ieee.org](mailto:wangkq@ieee.org)

Henggui Zhang: henggui.zhang@manchester.ac.uk

# Methods

## Development of *I_CaL_* Models

L-type calcium current (*I_CaL_*) equations were based on the Ten Tusscher and Panfilov (TP06) human ventricular cell model (Ten Tusscher and Panfilov, 2006). TP06 model was modified to incorporate the experimental data on the G1783C, wild-type (WT), P381S, M456I, A582D and R858H *CACNA1C* mutations (Fukuyama et al., 2014). These experimental data include (1) the current-voltage (I-V) relationships, (2) biophysical parameters for voltage-dependent activation kinetics, (3) biophysical parameters for voltage-dependent inactivation kinetics and (4) the inactivation time constant (Fukuyama et al., 2014).

To obtain the model parameters that reproduced the experimental data of the G1783C, WT, P381S, M456I, A582D and R858H *CACNA1C* mutations, we simulated the I-V curves by using the experimental protocol from Fukuyama *et al.* (Fukuyama et al., 2014). 300 ms pulses were applied from a holding potential of -70 mV to test potentials between -50 and +50 mV, in 10 mV increments. Biophysical parameters for the modified model were determined by fitting measured activation and inactivation data. Parameters then were optimized to account for the behavior of macroscopic currents compared with the experimental data and the original *I_CaL_* current of the TP06 human ventricular model (Ten Tusscher et al., 2004; Ten Tusscher and Panfilov, 2006).

*CACNA1C* mutations-induced changes in *I_CaL_* channel kinetics include *V_a,0.5_* the half-activation voltage, *S_a_*the slope of the steady-state activation of *I_CaL_* channel, *V_ina,0.5_* the half-inactivation voltage*, S_ina_*the slope of the steady-state inactivation of*I_CaL_* channel, *CSF* a scaling factor of the maximal conductance of *I_CaL_* and *TCSF* a scaling factor of the voltage-dependent time constant of inactivation (for details see **Supplementary Table S1**). Following the TP06 model, *I_CaL_* was described as

$$\boldsymbol{I}_{\boldsymbol{CaL}}\boldsymbol{=CSF}\boldsymbol{G}_{\boldsymbol{CaL}}\boldsymbol{df}\boldsymbol{f}_{\boldsymbol{2}}\boldsymbol{4}\frac{\left( \boldsymbol{V-15} \right)\boldsymbol{F}^{\boldsymbol{2}}}{\boldsymbol{RT}}\frac{\boldsymbol{0.25}\boldsymbol{Ca}_{\boldsymbol{SS}}\boldsymbol{e}^{\frac{\boldsymbol{2}\left( \boldsymbol{V-15} \right)\boldsymbol{F}}{\boldsymbol{RT}}}\boldsymbol{-}\boldsymbol{Ca}_{\boldsymbol{o}}}{\boldsymbol{e}^{\frac{\boldsymbol{2}\left( \boldsymbol{V-15} \right)\boldsymbol{F}}{\boldsymbol{RT}}}\boldsymbol{-1}}$$

$$\frac{\boldsymbol{dd}}{\boldsymbol{dt}}\boldsymbol{=}\frac{\boldsymbol{(}\boldsymbol{d}_{\boldsymbol{\infty}}\boldsymbol{-d)}}{\boldsymbol{\tau}_{\boldsymbol{d}}}$$

$$\boldsymbol{d}_{\boldsymbol{\infty}}\boldsymbol{=}\frac{\boldsymbol{1}}{\boldsymbol{1+}\boldsymbol{e}^{\boldsymbol{(}\boldsymbol{V}_{\boldsymbol{a,0.5}}\boldsymbol{-V)/}\boldsymbol{S}_{\boldsymbol{a}}}}$$

$$\frac{\boldsymbol{df}}{\boldsymbol{dt}}\boldsymbol{=}\frac{\boldsymbol{(}\boldsymbol{f}_{\boldsymbol{\infty}}\boldsymbol{-f)}}{\boldsymbol{TCSF\tau}_{\boldsymbol{f}}}$$

$$\boldsymbol{f}_{\boldsymbol{\infty}}\boldsymbol{=}\frac{\boldsymbol{1}}{\boldsymbol{1+}\boldsymbol{e}^{\boldsymbol{(V-}\boldsymbol{V}_{\boldsymbol{ina,0.5}}\boldsymbol{)/}\boldsymbol{S}_{\boldsymbol{ina}}}}$$

where *G_CaL_* is the maximal conductance of *I_CaL_* (μS/pF), *d* is the activation variable, *f* is the voltage-dependent inactivation variable and*τ_f_* is the voltage-dependent time constant of inactivation_._ For details of other equations please see (Ten Tusscher and Panfilov, 2006).

## Modeling calcium cycling

Calcium release flux (*I_rel_*) from sarcoplasmic reticulum (SR) ryanodine receptor (RyR2) was modeled with the combination of both calcium-induced-calcium release and SR calcium leak. The *I_rel_* model is equivalent to that used in the human model (Lascano et al., 2013; Liu et al., 2016; Bai et al., 2017). Equations for calcium dynamics:

$$\boldsymbol{I}_{\boldsymbol{rel}}\boldsymbol{=}\left( \boldsymbol{V}_{\boldsymbol{rel}}\boldsymbol{\cdot O+}\boldsymbol{V}_{\boldsymbol{sp}}\boldsymbol{\cdot R} \right)\boldsymbol{\cdot}\left( \left[ \boldsymbol{Ca}^{\boldsymbol{2+}} \right]_{\boldsymbol{SR}}\boldsymbol{-}\left[ \boldsymbol{Ca}^{\boldsymbol{2+}} \right]_{\boldsymbol{SS}} \right)$$

$$\boldsymbol{I}_{\boldsymbol{up}}\boldsymbol{=}\frac{\boldsymbol{V}_{\boldsymbol{maxup}}}{\boldsymbol{1+}\boldsymbol{K}_{\boldsymbol{up}}^{\boldsymbol{2}}\boldsymbol{/}{\boldsymbol{[}\boldsymbol{Ca}^{\boldsymbol{2+}}\boldsymbol{]}}_{\boldsymbol{i}}^{\boldsymbol{2}}}$$

$$\frac{\boldsymbol{d}\left[ \boldsymbol{Ca}^{\boldsymbol{2+}} \right]_{\boldsymbol{i}}}{\boldsymbol{dt}}\boldsymbol{=}\boldsymbol{-}\frac{\boldsymbol{I}_{\boldsymbol{bCa}}\boldsymbol{+}\boldsymbol{I}_{\boldsymbol{pCa}}\boldsymbol{-2}\boldsymbol{I}_{\boldsymbol{NCX}}}{\boldsymbol{2}\boldsymbol{V}_{\boldsymbol{c}}\boldsymbol{F}}\mathbf{+}\frac{\boldsymbol{V}_{\boldsymbol{sr}}}{\boldsymbol{V}_{\boldsymbol{c}}}\left( \boldsymbol{-}\boldsymbol{I}_{\boldsymbol{up}} \right)\boldsymbol{+}\boldsymbol{I}_{\boldsymbol{xfer}}$$

$$\frac{\boldsymbol{d}\left[ \boldsymbol{Ca}^{\boldsymbol{2}+} \right]_{\boldsymbol{SR}}}{\boldsymbol{dt}}\boldsymbol{=(}\boldsymbol{I}_{\boldsymbol{up}}\boldsymbol{-}\boldsymbol{I}_{\boldsymbol{rel}}\boldsymbol{)}$$

where *V_rel_* denotes rate constant of SR calcium release, *V_sp_* denotes rate constant of SR calcium leak, *O* denotes open state of RyR2, *R* denotes close state of RyR2, *[Ca^2+^]_SR_* denotes concentration of calcium ion in sub-cellular compartment SR (*mM*) and *[Ca^2+^]_SS_* denotes concentration of calcium ion in sub-cellular compartment dyadic cleft (*SS*) (*mM*). For details of other equations please see the TP06 model (Ten Tusscher and Panfilov, 2006).

## Pseudo-ECG simulation

According to the method of Gima and Rudy (Gima and Rudy, 2002), the pseudo-ECG was calculated as an integral of membrane potential between the extracellular position (*x*’, *y*’, *z’*) and the strand position(*x*, *y*, *z*).

$$\boldsymbol{\emptyset}_{\boldsymbol{e}}\left( \boldsymbol{x}^{\boldsymbol{'}}\boldsymbol{,}\boldsymbol{y}^{\boldsymbol{'}}\boldsymbol{,}\boldsymbol{z}^{\boldsymbol{'}} \right)\boldsymbol{=}\frac{\boldsymbol{\alpha}^{\boldsymbol{2}}\boldsymbol{\sigma}_{\boldsymbol{i}}}{\boldsymbol{4}\boldsymbol{\sigma}_{\boldsymbol{e}}}\int\left( \boldsymbol{-}\boldsymbol{\nabla}\boldsymbol{V}_{\boldsymbol{m}} \right)\boldsymbol{\cdot}\left[ \boldsymbol{\nabla}\frac{\boldsymbol{1}}{\boldsymbol{r}} \right]\boldsymbol{dx}$$

$$\boldsymbol{r=}\sqrt{\boldsymbol{(x-x')}^{\boldsymbol{2}}\boldsymbol{+}\boldsymbol{(y-y')}^{\boldsymbol{2}}\boldsymbol{+}\boldsymbol{(z-z')}^{\boldsymbol{2}}}$$

where*σ_i_* and *σ_e_* denote the intracellular and extracellular conductivities, respectively. *α* denotes the fiber radius and *r* denotes the distance between the extracellular position (*x*’, *y*’, *z’*) and the strand position(*x*, *y*, *z*). The extracellular position (*x*’, *y*’, *z’*) is 2 cm from the epicardial end of the transmural ventricular fiber.

## Model independence of the cellular electrophysiological consequences of *CACNA1C* Mutations

In addition to using the TP06 model (Ten Tusscher and Panfilov, 2006), we also used the O'Hara *et al.* (ORd) ventricular cell model (O'Hara et al., 2011) to examine the cellular electrophysiological alterations arising from *CACNA1C* mutations. According to the TP06 model, the same *I_CaL_* model was incorporated into the ORd endocardial (ENDO), midmyocardial (MCELL) and epicardial (EPI) cells, and transmural heterogeneity of ionic channel current density of *I_CaL_* was not considered in these simulations.

Action potential duration (APD), L-type calcium current (*I_CaL_*), calcium induced calcium release flux (*I_rel_*), the sodium-calcium exchanger current (*I_NCX_*), cytoplasmic calcium concentration (*[Ca^2+^]_i_*), junctional sarcoplasmic reticulum (SR) calcium concentration (*[Ca^2+^]_JSR_*) and network SR calcium concentration (*[Ca^2+^]_JSR_*) at a pacing frequency of 1Hz were gradually increased from the G1783C to WT, P381S, M456I and A582D, R858H conditions (**Figure S2**).

In **Figure S3**, we show the rate-dependent abbreviation of human ventricular APD for ENDO (**Figure S3**, **left column**), MCELL (**Figure S3**, **middle column**) and EPI (**Figure S3**, **right column**) cells under G1783C, wild-type (WT), P381S, M456I, A582D and R858H conditions. Pacing cycle lengths (PCL) of 500 ms (Black), 1000 ms (Red) and 2000 ms (Green) were used. However, afterdepolarizations were not predicted under the R858H condition. This can be attributed to the *I_rel_* model based on the Luo-Rudy model (Luo and Rudy, 1994) that cannot reproduce delayed afterdepolarizations (DADs) (Fink et al., 2011).

In **Figure S4**, we show action potential duration (APD) restitution (APDR) curves for ENDO (**Figure S4**, **left column**), MCELL (**Figure S4**, **middle column**) and EPI (**Figure S4**, **right column**) cells. Maximum slopes of APDR curves were gradually increased from G1783C to WT, P381S, M456I and A582D, R858H conditions, and did not exceed 1, suggesting stable excitation waves.

The simulations results support our original results: APD prolongation, SR calcium overload and steep APDR under the R858H condition.

# Legends for supporting figures

**Figure S1**. Simulated focal activity under the R858H condition at the pacing cycle length (PCL) of 500 ms in 1D midmyocardial (MCELL) cable. This is a part of the **Figure 9** (R858H) from 35000 ms to 35500 ms. Color mapping of membrane potential of cells along the 1D strand from -85.3 mV to -73 mV. Space runs from 0 mm to 24.75 mm.

**Figure S2.** Snapshots of transmural conduction of electrical waves in a 24.75🞨150 mm^2^ transmual ventricular sheet. Action potential propagation (*t* = 50 ms, 400 ms, 750 ms and 1000 ms) under G1783C, wild-type (WT), P381S, M456I, A582D and R858H conditions, respectively. The S1-S1 interval for initiating spiral waves was increased from 310 ms (G1783C) to 315 ms (WT), 320 ms (P381S), 335 ms (M456I), 340 ms (A582D) and 365 ms (R858H), respectively.

**Figure S3.** Snapshots of transmural conduction of electrical waves in a transmural ventricular slice. Action potential propagation (*t* = 10 ms, 350 ms, 440 ms, 520 ms and 990 ms) under G1783C, wild-type (WT), P381S, M456I, A582D and R858H conditions, respectively. The S1-S1 interval for initiating spiral waves was 310 ms (G1783C), 315 ms (WT), 320 ms (P381S), 325 ms (M456I), 330 ms (A582D) and 345 ms (R858H), respectively.

**Figure S4.** Dynamics of electrical waves in 3D human ventricles. Action potential propagation (*t* = 10 ms, 300 ms, 600 ms and 900 ms) under G1783C, wild-type (WT), P381S, M456I, A582D and R858H conditions, respectively. The S1-S1 interval for initiating spiral waves was 250 ms (G1783C), 300 ms (WT), 309 ms (P381S), 340 ms (M456I), 345 ms (A582D) and 348 ms (R858H), respectively. Snapshots for the anterior and posterior views were given.

**Figure S5.** Simulation of intracellular calcium handling and action potential for the O’Hara-Rudy dynamic (ORd) human ventricular cell model. For endocardial (ENDO, **left column**), midmyocardial (MCELL, **middle column**) and epicaridal (EPI, **right column**) cells, time courses of membrane potential (E_m_), the L-type calcium current (*I_CaL_*), the sodium-calcium exchanger current (*I_NCX_*), cytoplasmic calcium concentration (*[Ca^2+^]_i_*), junctional sarcoplasmic reticulum (SR) calcium concentration (*[Ca^2+^]_JSR_*) and network SR calcium concentration (*[Ca^2+^]_JSR_*) in the G1783C (Magenta), wild-type (WT, Black), P381S (Red), M456I (Green), A582D (Blue) and R858H (Cyan) conditions are shown. The pacing cycle length (PCL) used in these simulations is 1000 ms.

**Figure S6.** Simulated action potentials (E_m_) for the O’Hara-Rudy dynamic (ORD) human ventricular cell model at different pacing rates under G1783C, wild-type (WT), P381S, M456I, A582D and R858H conditions. Endocardial (ENDO, **left column**), midmyocardial (MCELL, **middle column**) and epicardial (EPI, **right column**) action potentials at pacing cycle length (PCL) of 500 (Black), 1000 (Red) and 2000 ms (Green), respectively.

**Figure S7**. Simulation of action potential duration (APD) restitution (APDR) curves for the O’Hara-Rudy dynamic (ORd) human ventricular cell model. APDR curves for the endocardial (ENDO, **left column**), midmyocardial (MCELL, **middle column**) and epicardial (EPI, **right column**) in the G1783C (Magenta), wild-type (WT, Black), P381S (Red), M456I (Green), A582D (Blue) and R858H (Cyan) conditions are shown. Maximum slopes of ENDO, MCELL and EPI APDR curves corresponding to the G1783C, WT, P381S, M456I, A582D and R858H conditions, respectively.

# Legends for supporting videos

**Video S1.** Conduction of excitation waves in a ventricular slice under the G1783C condition. The S1-S1 interval was 310 ms.

**Video S2.** Conduction of excitation waves in a ventricular slice under the wild-type (WT) condition. The S1-S1 interval was 315 ms.

**Video S3.** Conduction of excitation waves in a ventricular slice under the P381S condition. The S1-S1 interval was 320 ms.

**Video S4.** Conduction of excitation waves in a ventricular slice under the M456I condition. The S1-S1 interval was 325 ms.

**Video S5.** Conduction of excitation waves in a ventricular slice under the A582D condition. The S1-S1 interval was 330 ms.

**Video S6.** Conduction of excitation waves in a ventricular slice under the R858H condition. The S1-S1 interval was 345 ms.

**Video S7.** Conduction of excitation waves in 3D ventricles under the G1783C condition. The S1-S1 interval was 250 ms.

**Video S8.** Conduction of excitation waves in 3D ventricles under the wild-type (WT) condition. The S1-S1 interval was 300 ms.

**Video S9.** Conduction of excitation waves in 3D ventricles under the P381S condition. The S1-S1 interval was 309 ms.

**Video S10.** Conduction of excitation waves in 3D ventricles under the M456I condition. The S1-S1 interval was 340 ms.

**Video S11.** Conduction of excitation waves in 3D ventricles under the A582D condition. The S1-S1 interval was 345 ms.

**Video S12.** Conduction of excitation waves in 3D ventricles under the R858H condition. The S1-S1 interval was 348 ms.

# Legends for supporting tables

- **Supplementary Table S1**. Experimental (E) and simulated (S) changes of L-type calcium channel kinetics for G1783C, wild-type (WT), P381S, M456I, A582D and R858H conditions.

|  | E(G1783C) | S(G1783C) | E(WT) | S(WT) | E(P381S) | S(P381S) | E(M456I) | S(M456I) | E(A582D) | S(A582D) | E(R858H) | S(R858H) |
| --- | --- | --- | --- | --- | --- | --- | --- | --- | --- | --- | --- | --- |
| CSF | - | 0.92 | - | 1 | - | 1.05 | - | 1.12 | - | 1.33 | - | 1.43 |
| PDSF | 0.86 | 0.86 | 1 | 1 | 1.04 | 1.04 | 1.08 | 1.08 | 1.19 | 1.19 | 1.54 | 1.54 |
| *V_a,0.5_* ,mV | -1.4 | 5 | -1.4 | 5 | -2 | 4.4 | -2 | 4.4 | 0.67 | 6.07 | -3.7 | 2.7 |
| *S_a_* ,mV | 7.6 | 7.6 | 7.7 | 7.7 | 7.1 | 7.1 | 8.1 | 8.1 | 8.9 | 8.9 | 7.0 | 7.0 |
| *V_ina,0.5_* ,mV | -28.6 | -28.6 | -22.3 | -22.3 | -21.5 | -21.5 | -23.9 | -23.9 | -21.8 | -21.8 | -21.5 | -21.5 |
| *S_ina_* ,mV | -9.7 | -9.7 | -7.6 | -7.6 | -7.3 | -7.3 | -9.6 | -9.6 | -9.0 | -9.0 | -11.2 | -11.2 |
| TCSF | 1.11 | 1.11 | 1 | 1 | 1 | 1 | 1 | 1 | 1.43 | 1.43 | 0.88 | 0.88 |

*Note: CSF is a scaling factor of the maximal conductance of the L-type calcium current* (*I_CaL_*)*;* *PDSF is a scaling factor of the peak I_CaL_ density in current-voltage* (*I-V*) *relationships; V_a,0.5_ is the half-activation voltage and S_a_ is the slope of the steady-state activation of I_CaL_* *channel; V_ina,0.5_ is the half-inactivation voltage and S_ina_ is the slope of the steady-state inactivation of I_CaL_* *channel;* *TCSF is a scaling factor of the inactivation time-constant*.

- **Supplementary Table S2**. Changes in cytoplasmic calcium concentration amplitude (*[Ca^2+^]_i(m)_*), sarcoplasmic reticulum (SR) calcium content (*[Ca^2+^]_SR(m)_*) and the calcium induced calcium release flux amplitude (*I_rel_*_(m)_), action potential duration (APD_90_) under the G1783C, wild-type (WT), P381S, M456I, A582D and R858H conditions. Epicardial (EPI), midmyocardial (MCELL) and endocardial (ENDO) cells were considered.

|  | ENDO | | | | MCELL | | | | EPI | | | |
| --- | --- | --- | --- | --- | --- | --- | --- | --- | --- | --- | --- | --- |
|  | *[Ca^2+^]_i(m)_,*mM | *[Ca^2+^]_SR(m)_*,mM | *I_rel(m)_*,mM/ms | APD_90_,ms | *[Ca^2+^]_i(m)_,*mM | *[Ca^2+^]_SR(m)_*,mM | *I_rel(m)_*,mM/ms | APD_90_,ms | *[Ca^2+^]_i(m)_,*mM | *[Ca^2+^]_SR(m)_*,mM | *I_rel(m)_*,mM/ms | APD_90_,ms |
| G1783C | 3.6e-4 | 2.54 | 0.070 | 264.4 | 5.5e-4 | 3.13 | 0.104 | 359 | 3.7e-4 | 2.56 | 0.073 | 266 |
| WT | 4.0e-4 | 2.68 | 0.078 | 267.8 | 6.2e-4 | 3.32 | 0.114 | 364.2 | 4.2e-4 | 2.72 | 0.082 | 269.4 |
| P381S | 4.5e-4 | 2.82 | 0.086 | 271.8 | 6.9e-4 | 3.49 | 0.124 | 370.4 | 4.7e-4 | 2.88 | 0.090 | 273.4 |
| M456I | 4.9e-4 | 2.94 | 0.093 | 277 | 7.5e-4 | 3.63 | 0.131 | 379.6 | 5.1e-4 | 3.00 | 0.097 | 278.6 |
| A582D | 5.7e-4 | 3.17 | 0.105 | 282.6 | 8.5e-4 | 3.86 | 0.143 | 389.4 | 6.0e-4 | 3.24 | 0.110 | 284.6 |
| R858H | 7.0e-4 | 3.51 | 0.123 | 294 | 11e-4 | 4.25 | 0.159 | 410.8 | 7.5e-4 | 3.61 | 0.130 | 295.6 |

- **Supplementary Table S3**. The electrical differences of EPI-ENDO (between epicaridal and endocardial cells), EPI-M (between epicaridal and midmyocardial cells) and ENDO-M (between endocaridal and midmyocardial cells). For the G1783C, wild-type (WT), P381S, M456I, A582D and R858H conditions, electrical heterogeneities of cytoplasmic calcium concentration amplitude (*[Ca^2+^]_i(m)_*), sarcoplasmic reticulum (SR) calcium content (*[Ca^2+^]_SR(m)_*) and the calcium induced calcium release flux amplitude (*I_rel_*_(m)_) and action potential duration (APD_90_) were considered.

|  | [Ca^2+^]_i(m)_,mM | | | [Ca^2+^]_SR(m)_,mM | | | *I_rel_*_(m)_,mM/ms | | | APD_90_,ms | | |
| --- | --- | --- | --- | --- | --- | --- | --- | --- | --- | --- | --- | --- |
|  | ENDO-EPI | ENDO-M | EPI-M | ENDO-EPI | ENDO-M | EPI-M | ENDO-EPI | ENDO-M | EPI-M | ENDO-EPI | ENDO-M | EPI-M |
| G1783C | 1E-5 | 1.9E-4 | 1.8E-4 | 0.02 | 0.59 | 0.57 | 0.003 | 0.034 | 0.031 | 1.6 | 94.6 | 93 |
| WT | 2E-5 | 2.2E-4 | 2E-4 | 0.04 | 0.64 | 0.6 | 0.004 | 0.036 | 0.032 | 1.6 | 96.4 | 94.8 |
| P381S | 2E-5 | 2.4E-4 | 2.2E-4 | 0.06 | 0.67 | 0.61 | 0.004 | 0.038 | 0.034 | 1.6 | 98.6 | 97 |
| M456I | 2E-5 | 2.6E-4 | 2.4E-4 | 0.06 | 0.69 | 0.63 | 0.004 | 0.038 | 0.034 | 1.6 | 102.6 | 101 |
| A582D | 3E-5 | 2.8E-4 | 2.5E-4 | 0.07 | 0.69 | 0.62 | 0.005 | 0.038 | 0.033 | 2 | 106.8 | 104.8 |
| R858H | 5E-5 | 4E-4 | 3.5E-4 | 0.1 | 0.74 | 0.64 | 0.007 | 0.036 | 0.029 | 1.6 | 116.8 | 115.2 |

- **Supplementary Table S4**. Rate dependence of action potential duration (APD) in G1783C, wild-type (WT), P381S, M456I, A582D and R858H cells. APDs of endocaridal (ENDO), midmyocardial (MCELL) and epicardial (EPI) cells were given for each mutant *CACNA1C* at the different pacing cycle length (PCL).

|  | ENDO (APD_90_, ms) | | | MCELL (APD_90_, ms) | | | EPI (APD_90_, ms) | | |
| --- | --- | --- | --- | --- | --- | --- | --- | --- | --- |
|  | PCL=500 ms | PCL=1000 ms | PCL=2000 ms | PCL=500 ms | PCL=1000 ms | PCL=2000 ms | PCL=500 ms | PCL=1000 ms | PCL=2000 ms |
| G1783C | 237.82 | 264.4 | 267.2 | 297.62 | 359 | 384.2 | 236.62 | 266 | 271 |
| WT | 242.02 | 267.8 | 269 | 302.82 | 364.2 | 386.6 | 240.62 | 269.4 | 273.4 |
| P381S | 245.02 | 271.8 | 272.6 | 307.02 | 370.4 | 391.2 | 243.82 | 273.4 | 277.6 |
| M456I | 249.62 | 277 | 277.4 | 314.22 | 379.6 | 399.2 | 248.62 | 278.6 | 282.8 |
| A582D | 250.82 | 282.6 | 287.6 | 315.82 | 389.4 | 408.8 | 250.22 | 284.6 | 289.2 |
| R858H | 273.82 | 294 | 298 | 348.2 (DAD) | 410.8 | 425.2 | 252.02 | 295.6 | 298.4 |

- **Supplementary Table S5**. The maximum slopes of action potential duration restitution (APDR) curves for endocaridal (ENDO), midmyocardial (MCELL) and epicardial (EPI) cells under G1783C, wild-type (WT), P381S, M456I, A582D and R858H conditions.

|  | G1783C | WT | P381S | M456I | A582D | R858H |
| --- | --- | --- | --- | --- | --- | --- |
| ENDO | 1.02 | 1.08 | 1.1 | 1.15 | 1.15 | 1.35 |
| EPI | 1.9 | 1.6 | 1.5 | 1.45 | 1.45 | 1.5 |
| MCELL | 2 | 1.8 | 1.7 | 1.7 | 1.7 | 2 |

- **Supplementary Table S6**. QT interval (QT), T-wave width (T width), T-wave amplitude (T peak), repolarization time (RT), dispersion of repolarization (DOR), maximal absolute spatial gradient of APD (MSG) at the EPI-M (between epicaridal and midmyocardial cells) junction and the maximum PCL (MPCL) that produced 2:1 block in G1783C, wild-type (WT), P381S, M456I, A582D and R858H conditions.

|  | QT, ms | T width, ms | T peak, mV | RT, ms | DOR, ms | MSG, ms/mm | MPCL, ms |
| --- | --- | --- | --- | --- | --- | --- | --- |
| G1783C | 397.1 | 60 | 1.051 | 360.48 | 60.94 | 18 | 350 |
| WT | 398.5 | 59.9 | 1.053 | 363.6 | 61.3 | 18.1 | 352 |
| P381S | 401.2 | 59.9 | 1.056 | 367.34 | 62.2 | 18.5 | 354 |
| M456I | 408.2 | 61.7 | 1.075 | 374.84 | 64.12 | 18.9 | 356 |
| A582D | 415.9 | 63.9 | 1.087 | 381.14 | 65.64 | 19.2 | 365 |
| R858H | 425.7 | 64.5 | 1.104 | 393.32 | 67.97 | 19.5 | 370 |

# References

Bai, J., Yin, R., Wang, K., and Zhang, H. (2017). Mechanisms underlying the emergence of post-acidosis arrhythmia at the tissue level: A theoretical study. *Frontiers in Physiology* 8(195). doi: 10.3389/fphys.2017.00195.

Fink, M., Noble, P.J., and Noble, D. (2011). Ca2+-induced delayed afterdepolarizations are triggered by dyadic subspace Ca2+ affirming that increasing SERCA reduces aftercontractions. *American Journal of Physiology-Heart and Circulatory Physiology* 301(3)**,** H921-H935. doi: 10.1152/ajpheart.01055.2010.

Fukuyama, M., Wang, Q., Kato, K., Ohno, S., Ding, W.G., Toyoda, F., et al. (2014). Long QT syndrome type 8: novel CACNA1C mutations causing QT prolongation and variant phenotypes. *Europace* 16(12)**,** 1828-1837. doi: 10.1093/europace/euu063.

Gima, K., and Rudy, Y. (2002). Ionic Current Basis of Electrocardiographic Waveforms A Model Study. *Circulation Research* 90(8)**,** 889-896. doi: 10.1161/01.RES.0000016960.61087.86.

Lascano, E.C., Said, M., Vittone, L., Mattiazzi, A., Mundina-Weilenmann, C., and Negroni, J.A. (2013). Role of CaMKII in post acidosis arrhythmias: a simulation study using a human myocyte model. *Journal of Molecular and Cellular Cardiology* 60**,** 172-183. doi: 10.1016/j.yjmcc.2013.04.018.

Liu, H., Bai, J., Wang, K., Li, Q., and Yuan, Y. (2016). Simulation Study of Ventricular Arrhythmia in Post Acidosis. *Progress in Biochemistry and Biophysics* 43(7)**,** 716-724. doi: 10.16476/j.pibb.2016.0070.

Luo, C., and Rudy, Y. (1994). A dynamic model of the cardiac ventricular action potential. II. Afterdepolarizations, triggered activity, and potentiation. *Circulation research* 74(6)**,** 1097-1113.

O'Hara, T., Virág, L., Varró, A., and Rudy, Y. (2011). Simulation of the undiseased human cardiac ventricular action potential: model formulation and experimental validation. *PLoS Computational Biology* 7(5)**,** e1002061. doi: 10.1371/journal.pcbi.1002061.

Ten Tusscher, K.H., Noble, D., Noble, P.J., and Panfilov, A.V. (2004). A model for human ventricular tissue. *American Journal of Physiology-Heart and Circulatory Physiology* 286(4)**,** H1573-H1589. doi: 10.1152/ajpheart.00794.2003.

Ten Tusscher, K.H., and Panfilov, A.V. (2006). Alternans and spiral breakup in a human ventricular tissue model. *American Journal of Physiology-Heart and Circulatory Physiology* 291(3)**,** H1088-1100. doi: 10.1152/ajpheart.00109.2006.
